# Cell Stress Induces Upregulation of Osteopontin via the ERK Pathway in Type II Alveolar Epithelial Cells

**DOI:** 10.1371/journal.pone.0100106

**Published:** 2014-06-25

**Authors:** Aki Kato, Takafumi Okura, Chizuru Hamada, Seigo Miyoshi, Hitoshi Katayama, Jitsuo Higaki, Ryoji Ito

**Affiliations:** Department of Cardiology, Pulmonology, Hypertension & Nephrology, Ehime University Graduate School of Medicine, Toon, Ehime, Japan; University of Tokushima, Japan

## Abstract

Osteopontin (OPN) is a multifunctional protein that plays important roles in cell growth, differentiation, migration and tissue fibrosis. In human idiopathic pulmonary fibrosis and murine bleomycin-induced lung fibrosis, OPN is upregulated in type II alveolar epithelial cells (AEC II). However, the mechanism of OPN induction in AEC II is not fully understood. In this study, we demonstrate the molecular mechanism of OPN induction in AEC II and elucidate the functions of OPN in AEC II and lung fibroblasts. Human lung adenocarcinoma cells (A549) and mouse alveolar epithelial cells (MLE12), used as type II alveolar epithelial cell lines for in vitro assays, and human pulmonary alveolar epithelial cells (HPAEpiC) were treated with either bleomycin, doxorubicin or tunicamycin. The mechanism of OPN induction in these cells and its function as a pro-fibrotic cytokine on A549 and lung fibroblasts were analyzed. The DNA damaging reagents bleomycin and doxorubicin were found to induce OPN expression in A549, MLE12 and HPAEpiC. OPN expression was induced via activation of the extracellular signal-regulated protein kinase (ERK)-dependent signaling pathway in A549 and MLE12. The endoplasmic reticulum (ER) stress-inducing reagent tunicamycin induced *OPN* mRNA expression in A549, MLE12 and HPAEpiC, and *OPN* mRNA expression was induced via activation of the ERK-dependent signaling pathway in A549 and MLE12. Another ER stress-inducing reagent thapsigargin induced the expression of *OPN* mRNA as well as the subsequent production of OPN in A549 and MLE12. Furthermore, OPN promoted the proliferation of A549 and the migration of normal human lung fibroblasts. Inhibition of OPN by small interference RNA or neutralizing antibody suppressed both of these responses. The results of this study suggest that cell stress induces the upregulation of OPN in AEC II by signaling through the ERK pathway, and that upregulated OPN may play a role in fibrogenesis of the lung.

## Introduction

Idiopathic pulmonary fibrosis (IPF) is a progressive and often lethal lung disorder. The histopathological features of IPF are usual interstitial pneumonia, which consists of honeycombing, patchy fibrosis, fibroblastic foci and hyperplasia of type II pneumocytes [1]. The mechanism of IPF remains poorly understood. Previously, it was thought that chronic lung inflammation causes fibrogenesis and, eventually, fibrotic scarring. However, therapeutic strategies based on anti-inflammatory treatments or immunosuppressive approaches have not been effective in the treatment of IPF. Recent reports suggest that IPF is associated with abnormal repair of injured alveolar epithelium [2]. When alveolar epithelium is injured, type II alveolar epithelial cells (AEC II) undergo hyperplasia and become suppliers of fibrogenic cytokines that induce the proliferation and migration of lung fibroblasts [3]. Potential causes of alveolar epithelium injury include DNA damage and endoplasmic reticulum (ER) stress [4], [5].

Osteopontin (OPN) is a glycosylated phosphoprotein that contains an arginine-glycine-aspartate integrin binding domain. OPN was first identified as a bone matrix protein with an adhesive function owing to its integrin binding activity. Recently, OPN has been shown to be expressed in various tissues and to be upregulated under pathological as well as physiological conditions. Consequently, investigators have focused on the roles of OPN in the pathogenesis of various diseases and its pathological roles as a pro-inflammatory and pro-fibrotic cytokine [6], [7].

OPN is upregulated in the tissues of human IPF and murine bleomycin-induced lung fibrosis [7]–[9]. In normal lungs, OPN is mainly expressed in alveolar macrophages, whereas in human IPF and murine bleomycin-induced lung fibrosis, OPN is upregulated in hyperplastic AEC II [7]–[9]. Additionally, in murine bleomycin-induced lung fibrosis, OPN-deficient mice develop altered lung fibrosis characterized by dilated distal air space and decreased type I collagen expression compared to wild-type mice [9]. These reports suggest that the expression of OPN might be induced in AEC II of human IPF and murine bleomycin-induced lung fibrosis and that OPN may play a role in epithelial repair and regeneration. However, the detailed mechanism of OPN induction in AEC II is not fully understood. In this study, we elucidate the molecular mechanism of OPN induction in AEC II by bleomycin, doxorubicin and tunicamycin. We also demonstrate that upregulated OPN plays a crucial role in the proliferation of AEC II and the migration of lung fibroblasts.

## Materials and Methods

### Reagents and antibodies

Bleomycin and tunicamycin were purchased from Sigma-Aldrich (St. Louis, MO, USA). Doxorubicin and thapsigargin were purchased from Wako Pure Chemical Industries, Ltd. (Osaka, Japan). PD98059 and U0126 were purchased from Calbiochem (La Jolla, CA, USA). Anti-phospho-specific p44/42 mitogen-activated protein kinase (MAPK) (extracellular signal-regulated protein kinase1/2 (ERK1/2)), anti-ERK1/2, anti-phospho-specific stress-activated protein kinase (SAPK)/c-Jun NH_2_-terminal kinase (JNK), anti-SAPK/JNK, anti-phospho-specific p38 MAPK, anti-p38 MAPK, anti-β-actin, anti-immunoglobulin heavy-chain binding protein (BiP) antibodies and horseradish peroxidase (HRP)-conjugated secondary antibody were purchased from Cell Signaling Technology (Danvers, MA, USA). Recombinant human OPN was purchased from R&D Systems (Minneapolis, MN, USA). Anti-human OPN neutralizing antibody (anti-OPN IgG) and its nonspecific control antibody (control IgG) were purchased from R&D Systems.

### Cell culture

Human lung adenocarcinoma cells (A549) and mouse alveolar epithelial cells (MLE12) from American Type Culture Collection (Manassas, VA, USA) were cultured in Dulbecco's Modified Eagle Medium (DMEM) and DMEM/Ham's F-12, respectively, supplemented with 10% fetal bovine serum (FBS) and antibiotics (100 U/mL penicillin and 100 µg/mL streptomycin). These cells exhibit the cuboidal cell morphology of AEC II. Human pulmonary alveolar epithelial cells (HPAEpiC) from ScienCell Research Laboratories (Carlsbad, CA, USA) were cultured in alveolar epithelial cell medium (ScienCell Research Laboratories) with poly-L-lysine-coated culture vessels (Corning Incorporated, Tewksbury, MA, USA). Normal human lung fibroblasts (NHLFs) from Lonza (Walkersville, MD, USA) were cultured in DMEM supplemented with 10% FBS and antibiotics.

### Quantitative real-time *reverse transcription polymerase chain reaction* (qRT-PCR)

Total RNA was extracted with Isogen II reagent from Nippon Gene (Tokyo, Japan) according to the manufacturer's protocol. Complementary DNA (cDNA) was synthesized from total RNA with a first-strand cDNA synthesis kit, SuperScript VILO cDNA Synthesis Kit from Life Technologies (Carlsbad, CA, USA), according to the manufacturer's protocol. qRT-PCR was performed with a LightCycler 480 SYBR Green I Master from Roche (Basel, Switzerland) with the LightCycler 480 instrument (Roche). Cycle threshold values of *OPN* were normalized to those of *β-actin* (*ACTB*). Sequences of primers used for qRT-PCR were as follows: human *OPN*, 5′-AAGCGAGGAGTTGAATGGTGCAT-3′ (sense) and 5′-TGTGGGTTTCAGCACTCTGGTCA-3′ (antisense); human *ACTB*, 5′-CTGGAACGGTGAAGGTGACA-3′ (sense) and 5′-AAGGGACTTCCTGTAACAATGCA-3′ (antisense); mouse *Opn*, 5′-GTATTGCTTTTGCCTGTTTGG-3′ (sense) and 5′-TGAGCTGCCAGAATCAGTCACT-3′ (antisense); mouse *Actb*, 5′-GGCTGTATTCCCCTCCATCG-3′ (sense) and 5′-CCAGTTGGTAACAATGCCATGT-3′ (antisense). Each experiment was performed in triplicate and repeated at least three times.

### *Enzyme-linked immunosorbent assay* (ELISA)

Cells were lysed in M-PER Mammalian Protein Extraction Reagent (Thermo Fisher Scientific Inc., Walthem, MA, USA) and Halt Protease Inhibitor Single-Use Cocktail EDTA-Free (Thermo Fisher Scientific Inc.) was added at a concentration of 10 µg/mL. OPN concentrations in cell lysates and cell culture media were determined by ELISA, using the Human Osteopontin Assay Kit from Immuno-Biological Laboratories (Gunma, Japan) according to the manufacturer's protocol. All samples were prepared in duplicate and each experiment was repeated at least three times.

### Western blotting

Each sample was separated by sodium dodecyl sulphate-polyacrylamide gel electrophoresis, and subsequently transferred to polyvinylidene difluoride membranes. The membranes were blocked with Tris-buffered saline containing 0.1% Tween20 (TBS-T) and 0.1% Block Ace Powder from DS Pharma Biomedical (Osaka, Japan), followed by incubation with a primary antibody. After washing with TBS-T, the membranes were incubated with an HRP-conjugated secondary antibody and then washed with TBS-T again. Immunoreactive proteins were detected by enhanced chemiluminescence using the Amersham ECL Prime Western Blotting Detection Reagent from GE Healthcare (Little Chalfont, Buckinghamshire, UK). Each experiment was repeated at least three times.

### RNA interference

Small interference RNA (siRNA) against human *OPN* and control siRNA were purchased from Qiagen (Venlo, Netherlands). The transfection of siRNA was performed with a Lipofectamine RNAiMAX Reagent from Life Technologies according to the manufacturer's protocol.

### Cell proliferation assay

To quantitate cell proliferation, DNA replication was assessed using the Click-iT 5-ethynyl-2′-deoxyuridine (EdU) Alexa Fluor 488 HCS assay kit from Life Technologies, according to the manufacturer's protocol. Twenty-four h after the transfection of human *OPN* siRNA or control siRNA, A549 were reseeded into 6-well plates and exposed to 1 µg/mL bleomycin for 48 h. Thereafter, the A549 were incubated with EdU for an additional 15 h and then fixed with 4% paraformaldehyde in PBS (Wako Pure Chemical Industries, Ltd.) for 15 minutes at room temperature. The fixed A549 in each well were washed twice with 3% bovine serum albumin (BSA) in PBS and permeabilized with 0.5% Triton X-100 in PBS for 20 minutes at room temperature. The permeabilized A549 in each well were again washed twice with 3% BSA in PBS, and subsequently incubated with 480 µL of Click-iT reaction cocktail for 30 minutes at room temperature in the absence of light. Thereafter, the A549 were washed once with 3% BSA in PBS and once with PBS and then incubated with HCS NuclearMask Blue stain in PBS for 30 minutes at room temperature in the absence of light. After washing twice with PBS, the A549 were then visualized by pseudocoloring with the Floid Cell Imaging Station (Life Technologies). Proliferating cells incorporate EdU into newly synthesized DNA as a nucleoside analog of thymidine. Consequently, the nuclei of proliferating cells showed green fluorescence as EdU-positive nuclei after treatment with Alexa Fluor azide. The nuclei of all cells showed blue fluorescence following treatment with HCS NuclearMask Blue stain. Twelve fields per well were recorded with the Floid Cell Imaging Station and the number of positively-staining cells was counted. This experiment was performed in triplicate and repeated at least three times.

### Cell migration assay

Cell migration assay was performed using the xCELLigence Real-Time Cell Analyzer (RTCA) Dual-Plate (DP) instrument (Acea Biosciences, San Diego, CA, USA) which was placed at 37°C in a humidified 5% CO_2_ incubator. Cell migration was assessed using specifically designed 16-well plates with 8 µm pores (CIM-plate 16, Acea Biosciences). These plates are similar to conventional transwell plates but exhibit microelectrode sensors located on the underside of the membranes of the upper chamber. When cells migrate through the pores of the membrane and contact the electronic sensors, the impedance of the electronic sensors is increased. These changes in impedance are monitored in real-time as cell index. The membranes of the upper chamber were coated with 20 µg/mL fibronectin from Roche. This experiment was performed according to the manufacturer's protocol. NHLFs were cultured with DMEM supplemented with 0.1% FBS and antibiotics for 18 h to allow for serum starvation. These cells were then resuspended in DMEM supplemented with antibiotics (serum-free DMEM) and seeded into the upper chamber at 15,000 cells/well. Cell culture medium in which A549 in serum-free DMEM were exposed to 1 µg/mL bleomycin for 48 h was designated “Conditioned medium”. OPN concentration in the conditioned medium was determined by ELISA and was approximately 1.05 µg/mL. The lower chamber was filled with either conditioned medium +4 µg/mL control IgG, conditioned medium +4 µg/mL anti-OPN IgG, or serum-free DMEM +4 µg/mL control IgG. The cell index was monitored automatically every 15 minutes for at least 24 h with the xCELLigence RTCA DP instrument. In some cases, the serum-free DMEM supplemented with 1 µg/mL recombinant human OPN, at a concentration similar to that in the conditioned medium, was used. The lower chamber was filled with either serum-free DMEM+1 µg/mL recombinant human OPN+4 µg/mL control IgG, serum-free DMEM+1 µg/mL recombinant human OPN+4 µg/mL anti-OPN IgG, or serum-free DMEM+4 µg/mL control IgG. The cell index was monitored similarly. Data analysis was performed with RTCA software version 1.2. This experiment was performed in triplicate and repeated at least three times.

### Statistical analysis

Data from two subgroups were analyzed with the Mann-Whitney U test. Data among more than two subgroups were analyzed by ANOVA followed by the Tukey-Kramer test. All data were expressed as mean ± S.E. Differences were considered significant if P<0.05.

## Results

### Bleomycin-induced OPN expression in AEC II

**Figure 1:**
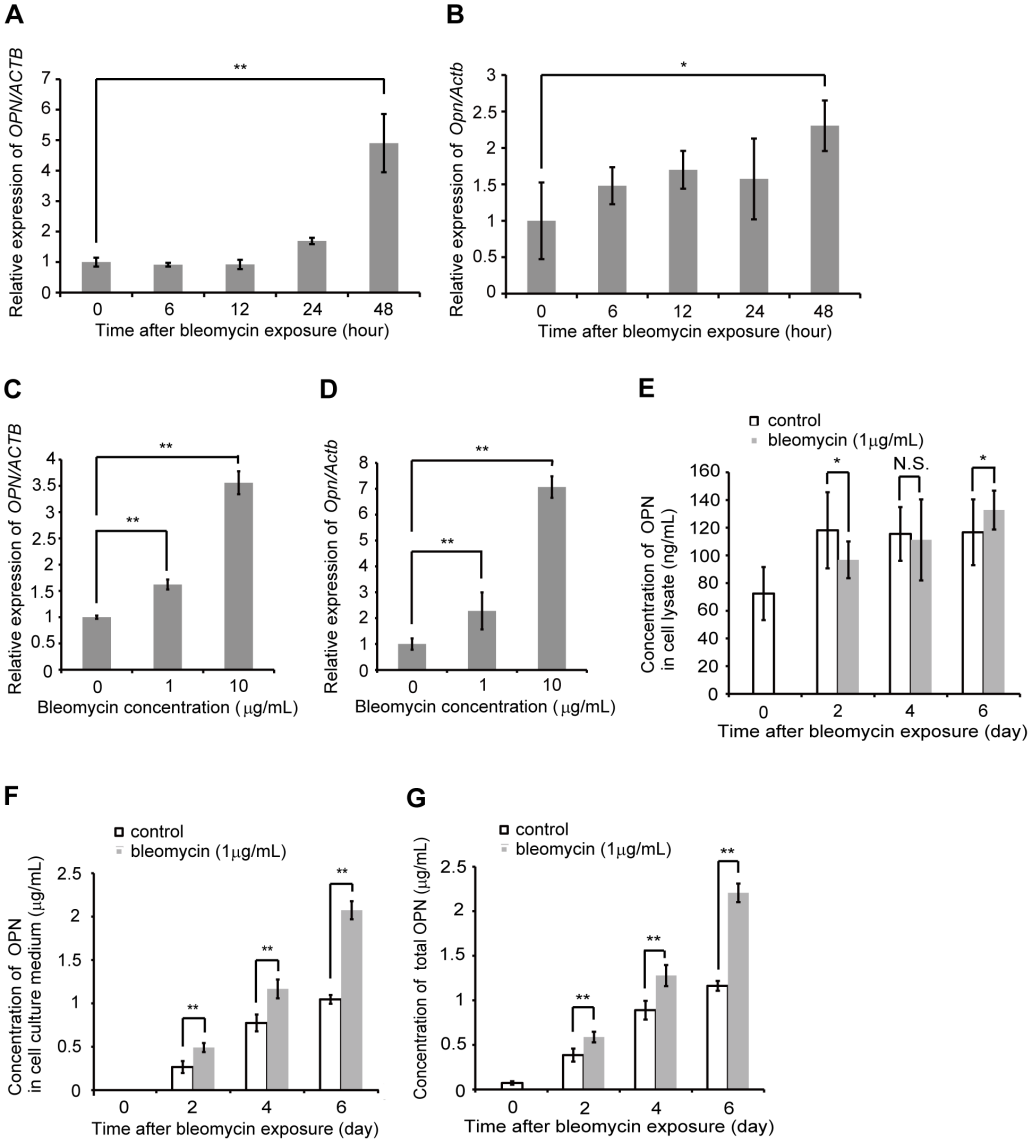
Effect of bleomycin on OPN expression in type II alveolar epithelial cell lines. (A) A549 were exposed to 1 µg/mL bleomycin for 0, 6, 12, 24 and 48 h. *OPN* mRNA was assessed by qRT-PCR. (B) MLE12 were exposed to 1 µg/mL bleomycin for 0, 6, 12, 24 and 48 h. *Opn* mRNA was assessed by qRT-PCR. (C) A549 were exposed to 0, 1 and 10 µg/mL bleomycin for 48 h. *OPN* mRNA was assessed by qRT-PCR. (D) MLE12 were exposed to 0, 1 and 10 µg/mL bleomycin for 48 h. *Opn* mRNA was assessed by qRT-PCR. (E, F and G) A549 were exposed to 1 µg/mL bleomycin. (E) The cells were lysed at 0, 2, 4 and 6 d. OPN concentrations in cell lysates were determined by ELISA. (F) The cell culture supernatants were collected at 0, 2, 4 and 6 d. OPN concentrations in cell culture media were determined by ELISA. (G) Total OPN concentrations in cell lysates and cell culture media were determined by ELISA. Data are presented as mean ± S.E. *, P<0.05; **, P<0.01.

We first sought to determine whether bleomycin would alter *OPN* mRNA expression in AEC II. After 48 h of exposure of A549 to 1 µg/mL bleomycin, the expression of *OPN* mRNA was elevated (Figure 1A). To confirm whether bleomycin would also alter *OPN* mRNA expression in other type II alveolar epithelial cell lines, MLE12 were exposed to 1 µg/mL bleomycin. Similar to A549, after 48 h of exposure to 1 µg/mL bleomycin, the expression of *Opn* mRNA was elevated in MLE12 (Figure 1B). Both A549 and MLE12 were subsequently exposed to 0, 1 and 10 µg/mL bleomycin for 48 h. Compared with 1 µg/mL, 10 µg/mL bleomycin induced higher *OPN* mRNA expression after 48 h (Figures 1C and 1D). Next, we sought to determine whether bleomycin would induce the production of OPN in A549. During 6 d of cultivation, OPN was upregulated spontaneously over time in both cell lysates and cell culture media. The accumulation of OPN in cell culture media was greater compared to that in cell lysates (Figures 1E and 1F). The addition of 1 µg/mL bleomycin reduced OPN in cell lysates at 2 d compared to the vehicle control (Figure 1E); however, it increased the total amount of OPN from cell lysates and cell culture media at 2, 4 and 6 d compared to the vehicle control (Figure 1G). These results suggest that treatment with bleomycin induces the expression of *OPN* mRNA and subsequent production of OPN in AEC II.

### Bleomycin-induced OPN expression via activation of the ERK-dependent signaling pathway

**Figure 2:**
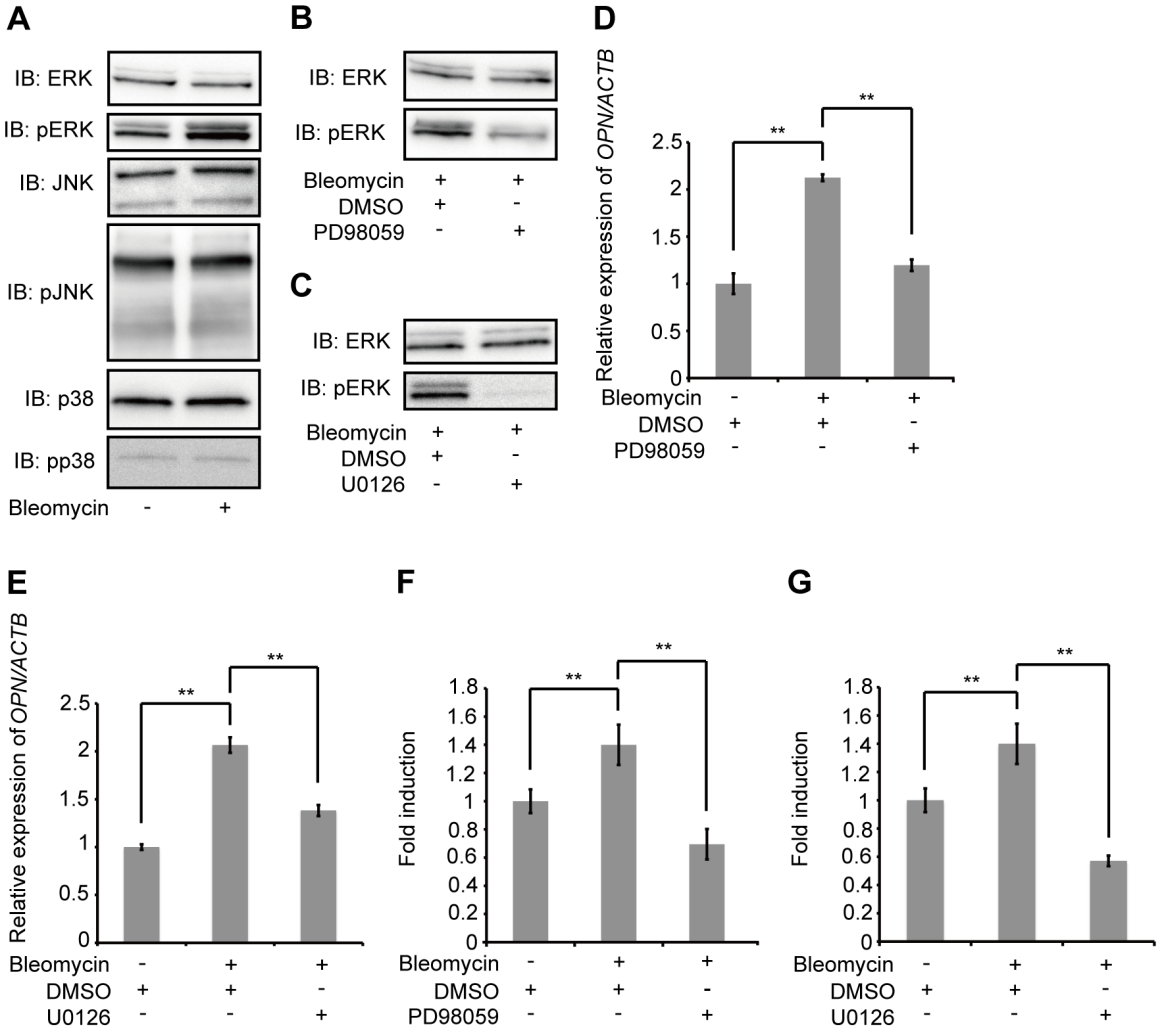
Molecular mechanism of OPN induction after exposure to bleomycin. (A) A549 were exposed to 10 µg/mL bleomycin or PBS for 24 h. Western blotting was used to detect phospho-specific ERK1/2, ERK1/2, phospho-specific SAPK/JNK, SAPK/JNK, phospho-specific p38 MAPK and p38 MAPK. (B) A549 were pretreated with PD98059 (50 µM) or DMSO for 1 h, and subsequently exposed to 10 µg/mL bleomycin for 48 h. Western blotting was used to detect phospho-specific ERK1/2 and ERK1/2. (C) A549 were pretreated with U0126 (50 µM) or DMSO for 1 h, and subsequently exposed to 10 µg/mL bleomycin for 48 h. Western blotting was used to detect phospho-specific ERK1/2 and ERK1/2. (D) A549 were pretreated with PD98059 (50 µM) or DMSO for 1 h, and subsequently exposed to 10 µg/mL bleomycin or PBS for 48 h. *OPN* mRNA was assessed by qRT-PCR. (E) A549 were pretreated with U0126 (50 µM) or DMSO for 1 h, and subsequently exposed to 10 µg/mL bleomycin or PBS for 48 h. *OPN* mRNA was assessed by qRT-PCR. (F) A549 were pretreated with PD98059 (50 µM) or DMSO for 1 h, and subsequently exposed to 10 µg/mL bleomycin or PBS for 48 h. OPN concentrations in cell culture media were determined by ELISA. (G) A549 were pretreated with U0126 (50 µM) or DMSO for 1 h, and subsequently exposed to 10 µg/mL bleomycin or PBS for 48 h. OPN concentrations in cell culture media were determined by ELISA. Data are presented as mean ± S.E. *, P<0.05; **, P<0.01.

The exposure of bovine endothelial cells or murine bronchoalveolar lavage fluid cells to bleomycin activates MAPKs [10], [11]. Furthermore, the activation of MAPKs induces the expression of OPN in rat cardiac fibroblasts and human periodontal ligament fibroblasts [12], [13]. Therefore, we sought to determine whether MAPKs are activated when A549 were exposed to bleomycin. ERK1/2 phosphorylation was induced in A549 after 24 h of exposure to 10 µg/mL bleomycin, without significant changes to total ERK1/2 protein levels. Moreover, bleomycin did not induce phosphorylation of SAPK/JNK or p38 MAPK in A549 (Figure 2A). To confirm these results, MLE12 were exposed to 10 µg/mL bleomycin. In MLE12, Erk1/2 phosphorylation was induced after 36 h of exposure, without significant changes to total Erk1/2 protein levels (Figure S1).

To clarify whether the ERK-dependent signaling pathway is responsible for bleomycin-mediated expression of OPN, we employed the use of two specific ERK1/2 inhibitors, PD98059 and U0126 [14], [15], and observed that at concentrations of 50 µM, both inhibited ERK1/2 phosphorylation after 48 h of bleomycin exposure, without significant changes to total ERK1/2 protein levels in A549 (Figures 2B and 2C) and MLE12 (Figures S2A and S2B). A549 were also pretreated with either PD98059 (50 µM) or U0126 (50 µM) for 1 h, after which the cells were exposed to either 10 µg/mL bleomycin or PBS for an additional 48 h. The presence of the inhibitors significantly reduced the expression of *OPN* mRNA (Figures 2D and 2E) as well as the accumulation of OPN in cell culture media (Figures 2F and 2G). To confirm these results, MLE12 were also pretreated with either PD98059 (50 µM) or U0126 (50 µM) for 1 h, after which the cells were exposed to either 10 µg/mL bleomycin or PBS for an additional 48 h. The presence of the inhibitors was found to significantly reduce expression of *Opn* mRNA in MLE12 (Figures S3A and S3B). These data suggest that bleomycin induces OPN expression via activation of the ERK-dependent signaling pathway in AEC II.

### Doxorubicin-induced OPN expression in AEC II via activation of the ERK-dependent signaling pathway

**Figure 3:**
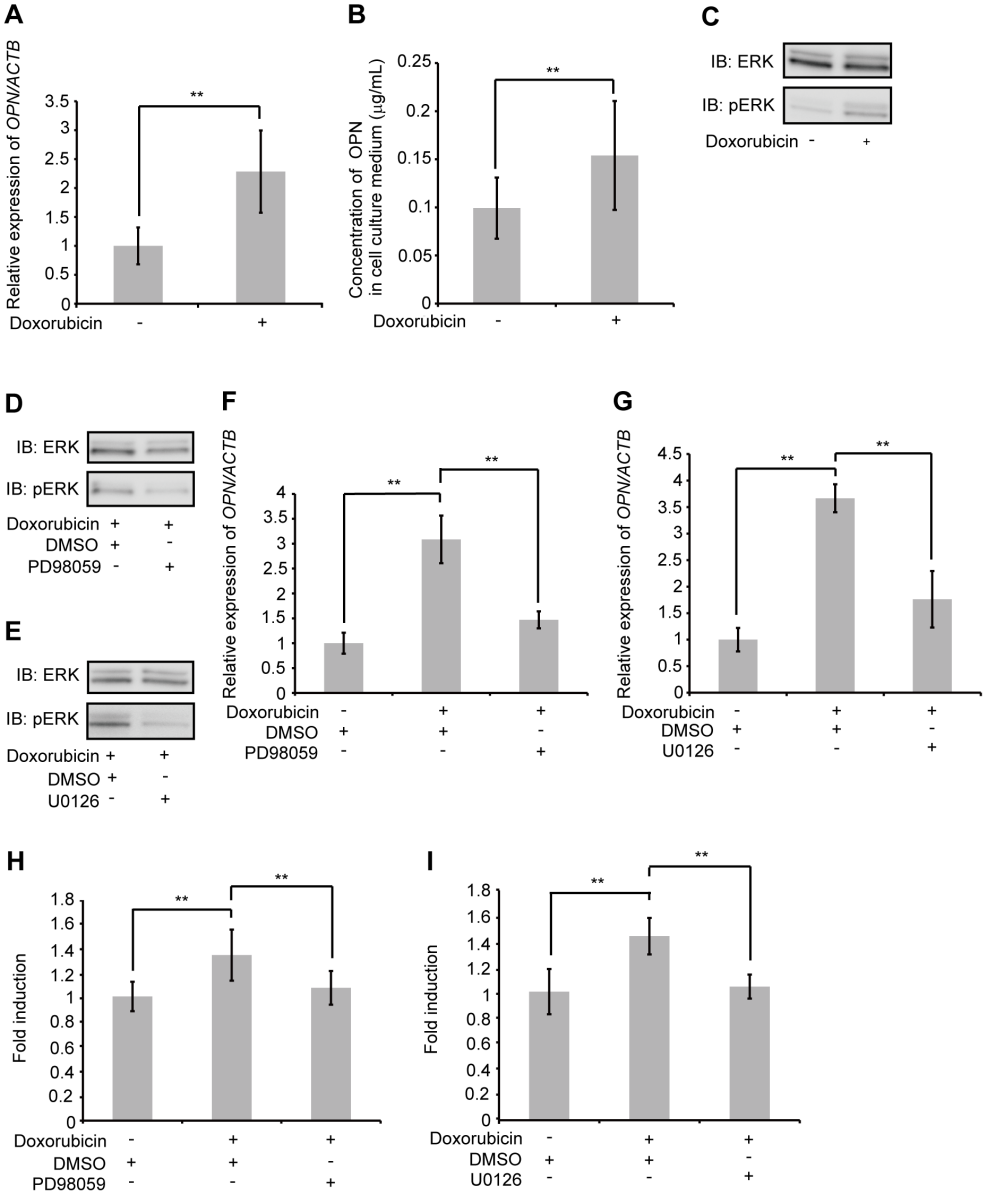
Effect of doxorubicin on OPN expression and molecular mechanism of OPN induction. (A and B) A549 were exposed to 50 nM doxorubicin or PBS for 48 h. (A) *OPN* mRNA was assessed by qRT-PCR. (B) OPN concentrations in cell culture media were determined by ELISA. (C) A549 were exposed to 50 nM doxorubicin or PBS for 24 h. Western blotting was used to detect phospho-specific ERK1/2 and ERK1/2. (D) A549 were pretreated with PD98059 (50 µM) or DMSO for 1 h, and subsequently exposed to 50 nM doxorubicin for 48 h. Western blotting was used to detect phospho-specific ERK1/2 and ERK1/2. (E) A549 were pretreated with U0126 (50 µM) or DMSO for 1 h, and subsequently exposed to 50 nM doxorubicin for 48 h. Western blotting was used to detect phospho-specific ERK1/2 and ERK1/2. (F) A549 were pretreated with PD98059 (50 µM) or DMSO for 1 h, and subsequently exposed to 50 nM doxorubicin or PBS for 48 h. *OPN* mRNA was assessed by qRT-PCR. (G) A549 were pretreated with U0126 (50 µM) or DMSO for 1 h, and subsequently exposed to 50 nM doxorubicin or PBS for 48 h. *OPN* mRNA was assessed by qRT-PCR. (H) A549 were pretreated with PD98059 (50 µM) or DMSO for 1 h, and subsequently exposed to 50 nM doxorubicin or PBS for 48 h. OPN concentrations in cell culture media were determined by ELISA. (I) A549 were pretreated with U0126 (50 µM) or DMSO for 1 h, and subsequently exposed to 50 nM doxorubicin or PBS for 48 h. OPN concentrations in cell culture media were determined by ELISA. Data are presented as mean ± S.E. *, P<0.05; **, P<0.01.

Bleomycin causes cellular injury through DNA strand breaks. To confirm whether cellular injury as a result of DNA damage would induce OPN expression in AEC II, we investigated the use of another reagent that causes cellular injury via inhibition of DNA topoisomerase, namely doxorubicin [16]. A549 and MLE12 were exposed to 50 nM doxorubicin. After 48 h of exposure, doxorubicin elevated the expression of *OPN* mRNA in both A549 (Figure 3A) and MLE12 (Figure S4), and resulted in increased accumulation of OPN in A549 cell culture media (Figure 3B). These data suggest that treatment with doxorubicin also induces the expression of *OPN* mRNA as well as the subsequent production of OPN in AEC II.

Next, we examined whether exposure of AEC II to doxorubicin can also induce activation of the ERK-dependent signaling pathway. ERK1/2 phosphorylation was induced in A549 after 24 h of exposure to 50 nM doxorubicin, without significant changes to total ERK1/2 protein levels (Figure 3C). To confirm these results, MLE12 were exposed to 50 nM doxorubicin. In MLE12, Erk1/2 phosphorylation was induced after 36 h of exposure, without significant changes to total Erk1/2 protein levels (Figure S5).

To determine whether the ERK-dependent signaling pathway was responsible for doxorubicin-mediated expression of OPN, we exposed both cell lines to 50 nM doxorubicin for 48 h after treatment with either PD98059 (50 µM) or U0126 (50 µM), and found that the ERK1/2 phosphorylation was inhibited without significant changes to total ERK1/2 protein levels in both A549 (Figures 3D and 3E) and MLE12 (Figures S6A and S6B). A549 and MLE12 were also pretreated with either PD98059 (50 µM) or U0126 (50 µM) for 1 h, prior to exposure to 50 nM doxorubicin or PBS for an additional 48 h. The presence of these inhibitors significantly reduced the expression of *OPN* mRNA in both A549 (Figures 3F and 3G) and MLE12 (Figures S7A and S7B), as well as the accumulation of OPN in A549 cell culture media (Figures 3H and 3I). These data suggest that DNA damage caused not only by bleomycin, but also by doxorubicin, activates the ERK-dependent signaling pathway in AEC II and induces OPN expression.

### Tunicamycin-induced *OPN* mRNA expression in AEC II via activation of the ERK-dependent signaling pathway

**Figure 4:**
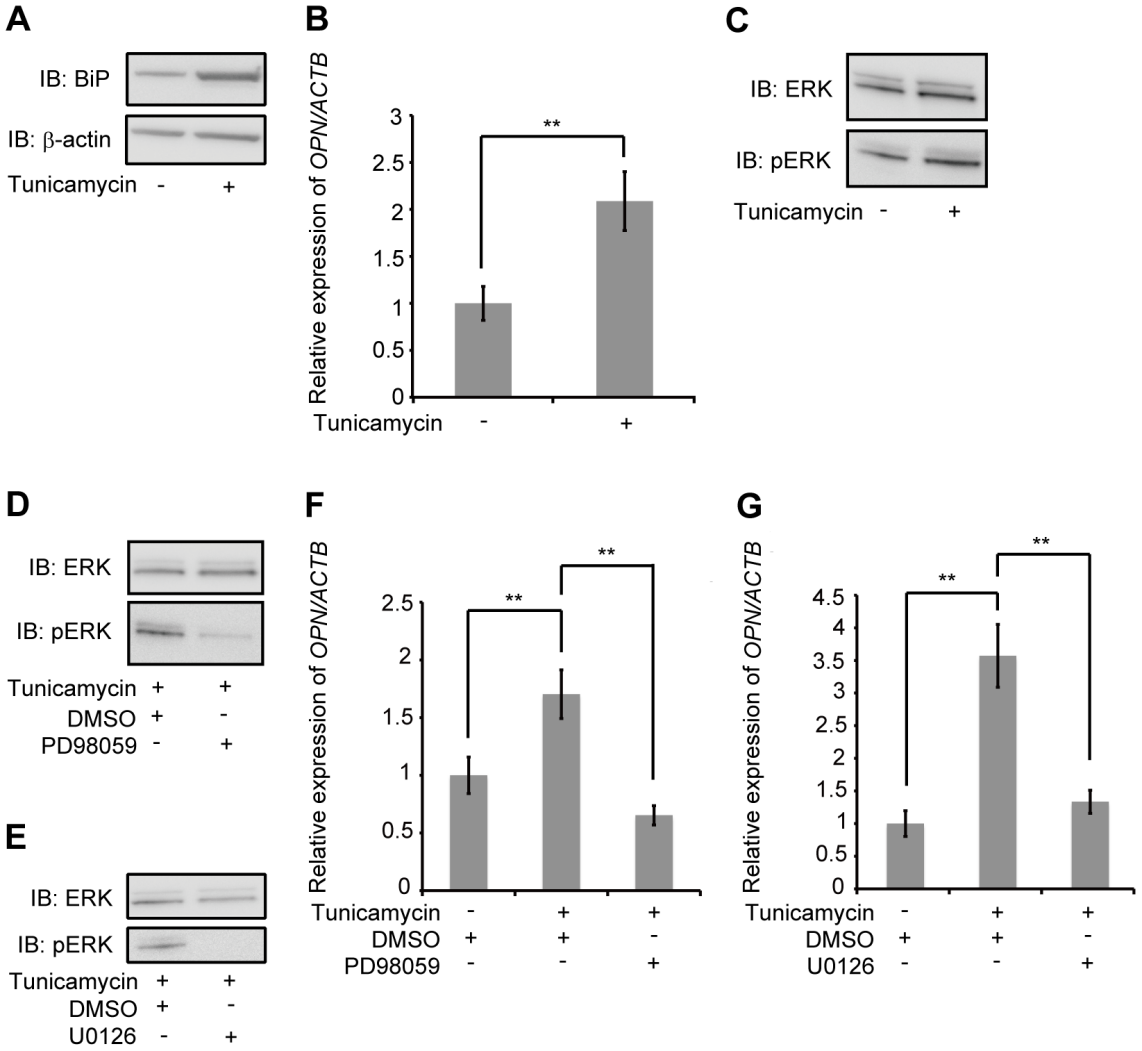
Effect of tunicamycin on OPN expression and molecular mechanism of OPN induction. (A) A549 were exposed to 0.5 µg/mL tunicamycin or DMSO for 48 h. Western blotting was used to detect BiP and β-actin. (B) A549 were exposed to 0.5 µg/mL tunicamycin or DMSO for 48 h. *OPN* mRNA was assessed by qRT-PCR. (C) A549 were exposed to 0.5 µg/mL tunicamycin or DMSO for 24 h. Western blotting was used to detect phospho-specific ERK1/2 and ERK1/2. (D) A549 were pretreated with PD98059 (50 µM) or DMSO for 1 h, and subsequently exposed to 0.5 µg/mL tunicamycin for 48 h. Western blotting was used to detect phospho-specific ERK1/2 and ERK1/2. (E) A549 were pretreated with U0126 (50 µM) or DMSO for 1 h, and subsequently exposed to 0.5 µg/mL tunicamycin for 48 h. Western blotting was used to detect phospho-specific ERK1/2 and ERK1/2. (F) A549 were pretreated with PD98059 (50 µM) or DMSO for 1 h, and subsequently exposed to 0.5 µg/mL tunicamycin or DMSO for 48 h. *OPN* mRNA was assessed by qRT-PCR. (G) A549 were pretreated with U0126 (50 µM) or DMSO for 1 h, and subsequently exposed to 0.5 µg/mL tunicamycin or DMSO for 48 h. *OPN* mRNA was assessed by qRT-PCR. Data are presented as mean ± S.E. *, P<0.05; **, P<0.01.

ER stress is associated with familial or sporadic IPF [17], [18]. Therefore, we examined the relationship between ER stress and OPN expression. We used tunicamycin as an ER-stress inducer [19] and determined the expression of BiP protein as a marker of ER stress [20]. A549 and MLE12 were exposed to 0.5 µg/mL and 0.025 µg/mL tunicamycin, respectively. After 48 h, tunicamycin upregulated BiP protein levels and induced the expression of *OPN* mRNA in both A549 (Figures 4A and 4B) and MLE12 (Figures S8 and S9). These data indicate that treatment with tunicamycin induces *OPN* mRNA expression in AEC II. In contrast to bleomycin and doxorubicin exposure, however, the accumulation of OPN in A549 cell culture media was not increased after exposure to tunicamycin (data not shown).

Subsequently, we examined whether the exposure of AEC II to tunicamycin induced the activation of the ERK-dependent signaling pathway. ERK1/2 phosphorylation was induced in A549 after 24 h of exposure to 0.5 µg/mL tunicamycin, without significant changes to total ERK1/2 protein levels (Figure 4C). To confirm these results, MLE12 were exposed to 0.025 µg/mL tunicamycin. In MLE12, Erk1/2 phosphorylation was induced after 36 h of exposure, without significant changes to total Erk1/2 protein levels (Figure S10).

Further, we sought to determine whether the ERK-dependent signaling pathway was responsible for tunicamycin-mediated expression of *OPN* mRNA. After 48 h exposure to tunicamycin, both PD98059 (50 µM) and U0126 (50 µM) were found to inhibit ERK1/2 phosphorylation without significant changes to total ERK1/2 protein levels in A549 (Figures 4D and 4E) and MLE12 (Figures S11A and S11B). A549 were pretreated with PD98059 (50 µM) or U0126 (50 µM) for 1 h, prior to exposure to 0.5 µg/mL tunicamycin or DMSO for an additional 48 h. These inhibitors significantly reduced the expression of *OPN* mRNA (Figures 4F and 4G). Similar results were obtained when MLE12 pretreated with PD98059 (50 µM) or U0126 (50 µM) for 1 h were subsequently exposed to 0.025 µg/mL tunicamycin (Figures S12A and S12B). Taken together, these results suggest that tunicamycin-induced ER stress also activates the ERK-dependent signaling pathway in AEC II, and induces the expression of *OPN* mRNA.

### Thapsigargin-induced OPN expression in AEC II

**Figure 5:**
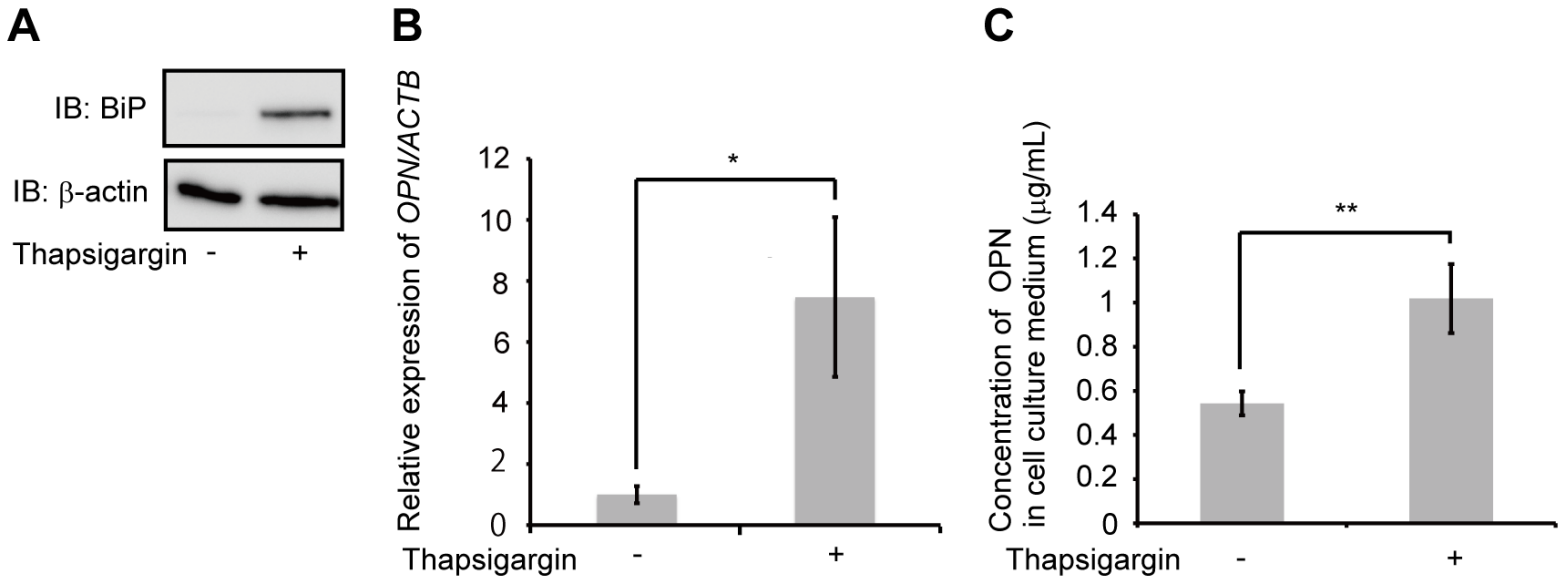
Effect of thapsigargin on OPN expression. (A) A549 were exposed to 10 nM thapsigargin or DMSO for 48 h. Western blotting was used to detect BiP and β-actin. (B and C) A549 were exposed to 10 nM thapsigargin or DMSO for 48 h. (B) *OPN* mRNA was assessed by qRT-PCR. (C) OPN concentrations in cell culture media were determined by ELISA. Data are presented as mean ± S.E. *, P<0.05; **, P<0.01.

Treatment with tunicamycin induces the expression of *OPN* mRNA; however, it does not increase the accumulation of OPN in A549 cell culture media. To confirm the relationship between ER stress and OPN expression, we investigated the use of another reagent that induces ER stress, namely thapsigargin. Thapsigargin induces ER stress by inhibiting Ca^2+^-ATPase in ER [21]. A549 and MLE12 were exposed to 10 nM and 5 nM thapsigargin, respectively. After 48 h, thapsigargin upregulated BiP protein levels and induced the expression of *OPN* mRNA in both A549 (Figures 5A and 5B) and MLE12 (Figures S13 and S14). Subsequently, we sought to determine whether thapsigargin would induce the production of OPN in A549 cell culture media. The accumulation of OPN in A549 cell culture media was increased after 48 h exposure to thapsigargin (Figure 5C). These data indicate that thapsigargin induces the expression of *OPN* mRNA and subsequent production of OPN in AEC II.

### Knockdown of OPN suppresses the proliferation of AEC II

**Figure 6:**
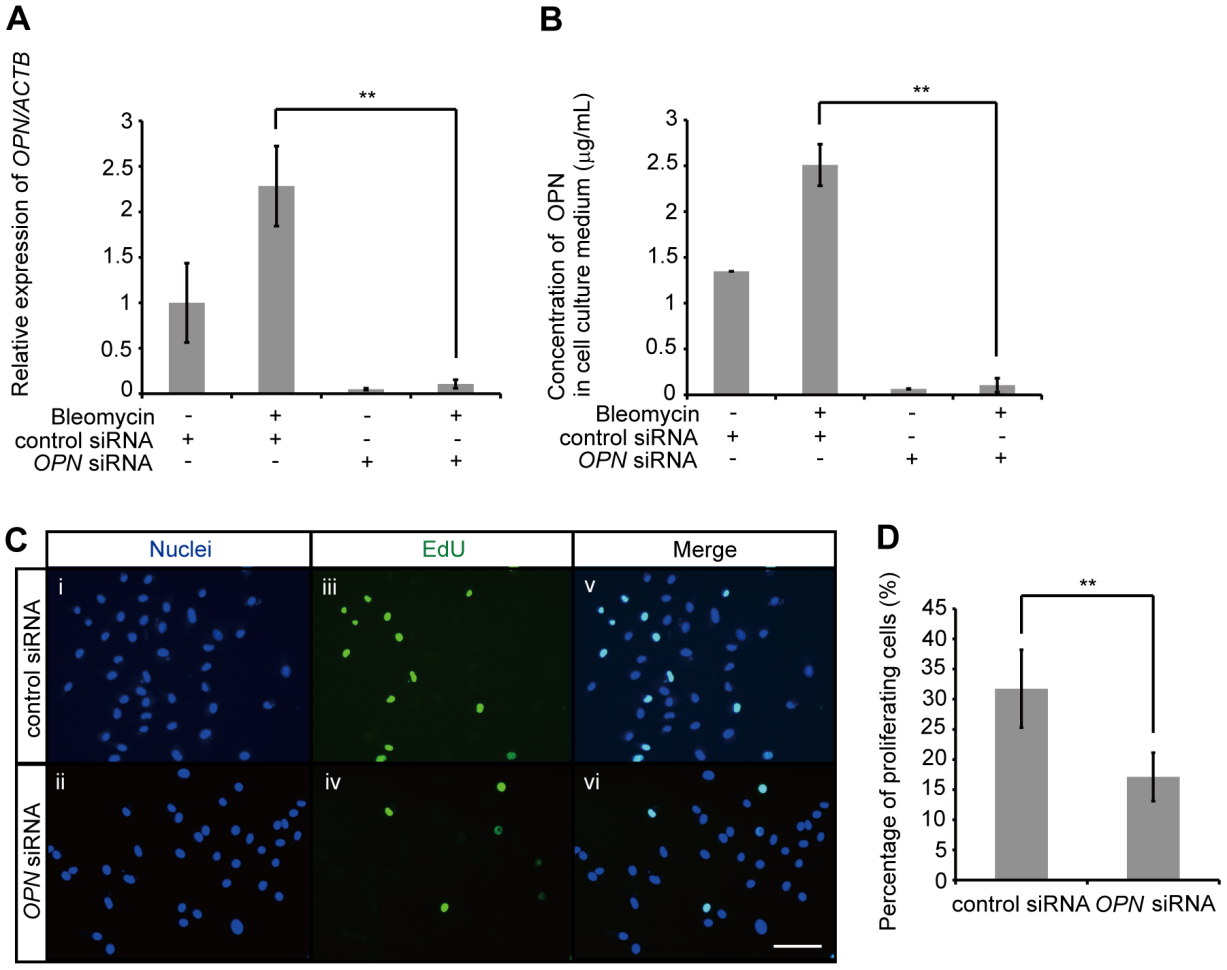
Effect of OPN on the proliferation of A549. (A and B) A549 were transfected with 20 nM human *OPN* siRNA or control siRNA. Twenty-four h after transfection, A549 were incubated for 48 h in the presence of 1 µg/mL bleomycin or PBS. (A) *OPN* mRNA was assessed by qRT-PCR. (B) OPN concentrations in cell culture media were determined by ELISA. (C and D) A549 were transfected with 20 nM human *OPN* siRNA or control siRNA. Twenty-four h after transfection, A549 were exposed to 1 µg/mL bleomycin for 48 h and then incubated with EdU for an additional 15 h. A549 were stained with Alexa Fluor 488 azide and HCS NuclearMask Blue stain. The cells were visualized by pseudocoloring with the Floid Cell Imaging Station. (C) Nuclei (i, ii) show representative photomicrographs of all cells. EdU (iii, iv) show representative photomicrographs of proliferating cells. Merge (v, vi) show representative photomicrographs of merged images. Bar: 100 µm. (D) Twelve fields per well were recorded and the number of positively-staining cells was counted. The percentages of proliferating A549 that were transfected with either human *OPN* siRNA or control siRNA are shown. Data are presented as mean ± S.E. *, P<0.05; **, P<0.01.

To assess the function of upregulated OPN in AEC II, human *OPN* siRNA was transfected into A549 at 20 nM. The expression of *OPN* mRNA (Figure 6A) and accumulation of OPN in cell culture media (Figure 6B) were significantly suppressed 3 d after transfection, regardless of the presence or absence of bleomycin exposure. Twenty-four h after the transfection of human *OPN* siRNA or control siRNA, A549 were exposed to 1 µg/mL bleomycin for 48 h. Thereafter, A549 were incubated with EdU for an additional 15 h. The nuclei of all cells showed blue fluorescence after treatment with HCS NuclearMask Blue stain (Figure 6C i and ii). Proliferating cells incorporate EdU into newly synthesized DNA as a nucleoside analog of thymidine. Consequently, the nuclei of proliferating cells showed green fluorescence as EdU-positive nuclei after treatment with Alexa Fluor azide (Figure 6C iii and iv). Twelve fields per well were recorded with the Floid Cell Imaging Station and the number of positively-staining cells was counted. In A549 transfected with human *OPN* siRNA, the percentage of proliferating cells was significantly decreased compared with those transfected with control siRNA (Figure 6D). This suggests that the expression of OPN by AEC II promotes self-proliferation in an autocrine manner.

### OPN protein in cell culture medium promotes the migration of lung fibroblasts

**Figure 7:**
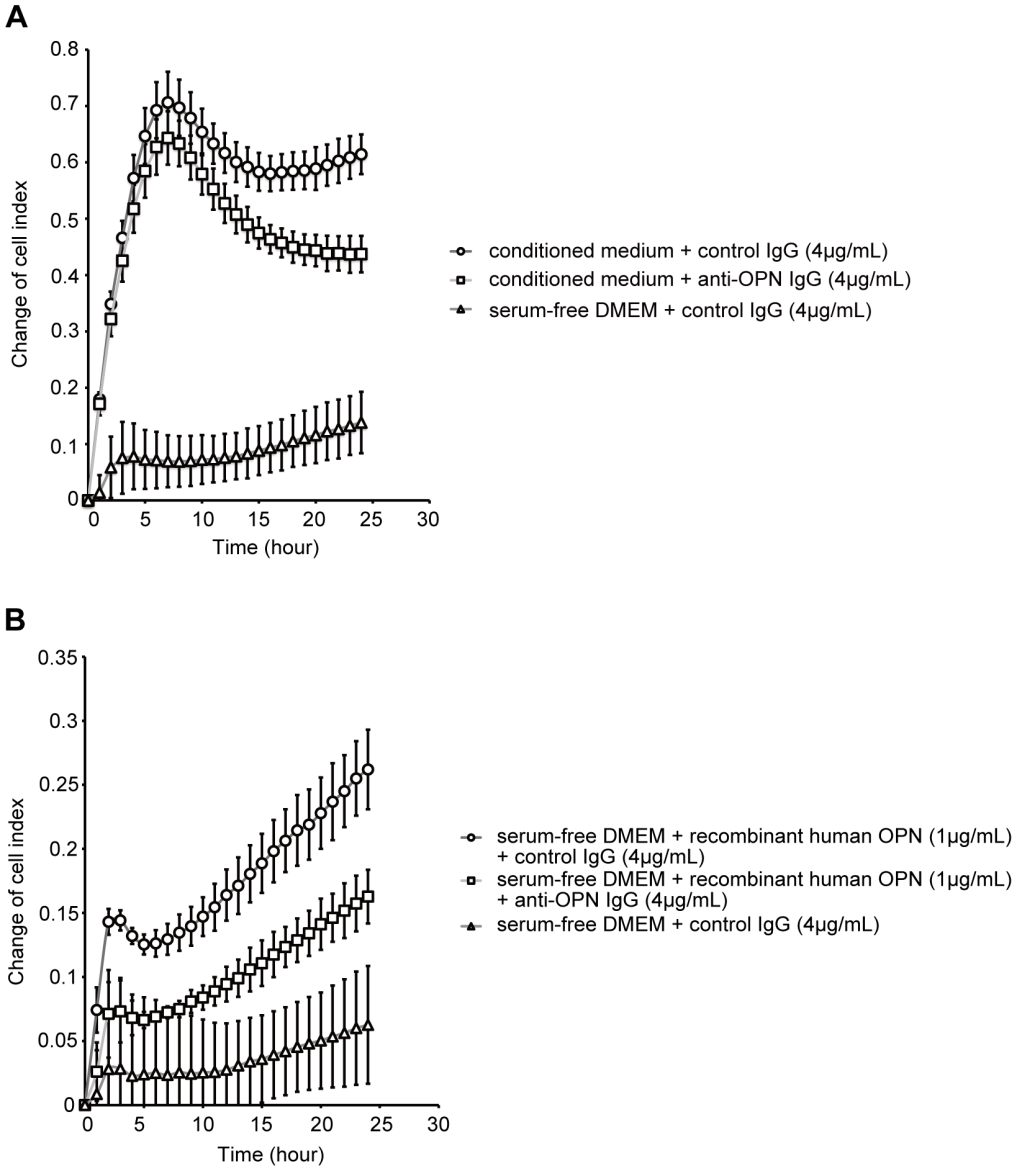
Effect of OPN on the migration of lung fibroblasts. (A and B) The migration of NHLFs was assessed with a xCELLigence RTCA DP instrument. Serum-starved NHLFs were resuspended in serum-free DMEM and seeded into the upper chamber at 15,000 cells/well. (A) Conditioned medium was made by exposing A549 to 1 µg/mL bleomycin in serum-free DMEM for 48 h. The lower chamber was filled with either conditioned medium +4 µg/mL control IgG, conditioned medium +4 µg/mL anti-OPN IgG, or serum-free DMEM+4 µg/mL control IgG. The cell index was monitored automatically every 15 minutes for at least 24 h. The vertical axis shows the change of cell index from the time when the CIM-plate 16 containing NHLFs was set into the xCELLigence RTCA DP instrument. (B) The lower chamber was filled with either serum-free DMEM+1 µg/mL recombinant human OPN+4 µg/mL control IgG, serum-free DMEM+1 µg/mL recombinant human OPN+4 µg/mL anti-OPN IgG, or serum-free DMEM+4 µg/mL control IgG. The cell index was monitored automatically every 15 minutes for at least 24 h. The vertical axis shows the change of cell index from the time when the CIM-plate 16 containing NHLFs was set into the xCELLigence RTCA DP instrument. Data are presented as mean ± S.E. *, P<0.05; **, P<0.01.

The migration of lung fibroblasts into the alveolar space is important for the evolution of intra-alveolar fibrosis, a step in the pathogenesis of IPF [22]. We assessed the effect of OPN on the migration of lung fibroblasts. First, we generated conditioned medium by exposing A549 to 1 µg/mL bleomycin in serum-free DMEM for 48 h. The concentration of OPN in the conditioned medium was approximately 1.05 µg/mL as determined by ELISA. NHLFs were cultured with DMEM supplemented with 0.1% FBS and antibiotics for 18 h for serum starvation. These cells were resuspended in serum-free DMEM and seeded into the upper chamber at 15,000 cells/well. The lower chamber was filled with either conditioned medium +4 µg/mL control IgG, conditioned medium +4 µg/mL anti-OPN IgG, or serum-free DMEM+4 µg/mL control IgG. The cell index was monitored automatically every 15 minutes for at least 24 h with the xCELLigence RTCA DP instrument. NHLFs migrated toward the conditioned medium after 24 h (conditioned medium + control IgG vs. serum-free DMEM + control IgG: 0.61±0.03 vs. 0.13±0.05, P<0.01). Moreover, the migration of NHLFs was significantly suppressed by anti-human OPN neutralizing antibody (conditioned medium + control IgG vs. conditioned medium + anti-OPN IgG: 0.61±0.03 vs. 0.43±0.03, P<0.01) (Figure 7A). In some cases, the lower chamber was filled with either serum-free DMEM+1 µg/mL recombinant human OPN+4 µg/mL control IgG, serum-free DMEM+1 µg/mL recombinant human OPN+4 µg/mL anti-OPN IgG, or serum-free DMEM+4 µg/mL control IgG. The cell index was monitored similarly. We also found that after 24 h, NHLFs migrated toward serum-free DMEM to which 1 µg/mL recombinant human OPN was added (serum-free DMEM + recombinant human OPN + control IgG vs. serum-free DMEM + control IgG: 0.26±0.03 vs. 0.06±0.04, P<0.01). The migration of NHLFs was significantly suppressed by anti-OPN IgG (serum-free DMEM + recombinant human OPN + control IgG vs. serum-free DMEM + recombinant human OPN + anti-OPN IgG: 0.26±0.03 vs. 0.16±0.02, P<0.05) (Figure 7B). These results suggest that the OPN produced in cell culture medium by AEC II after bleomycin treatment promotes the migration of lung fibroblasts.

### Bleomycin-, doxorubicin- and tunicamycin-induced OPN expression in HPAEpiC

**Figure 8:**
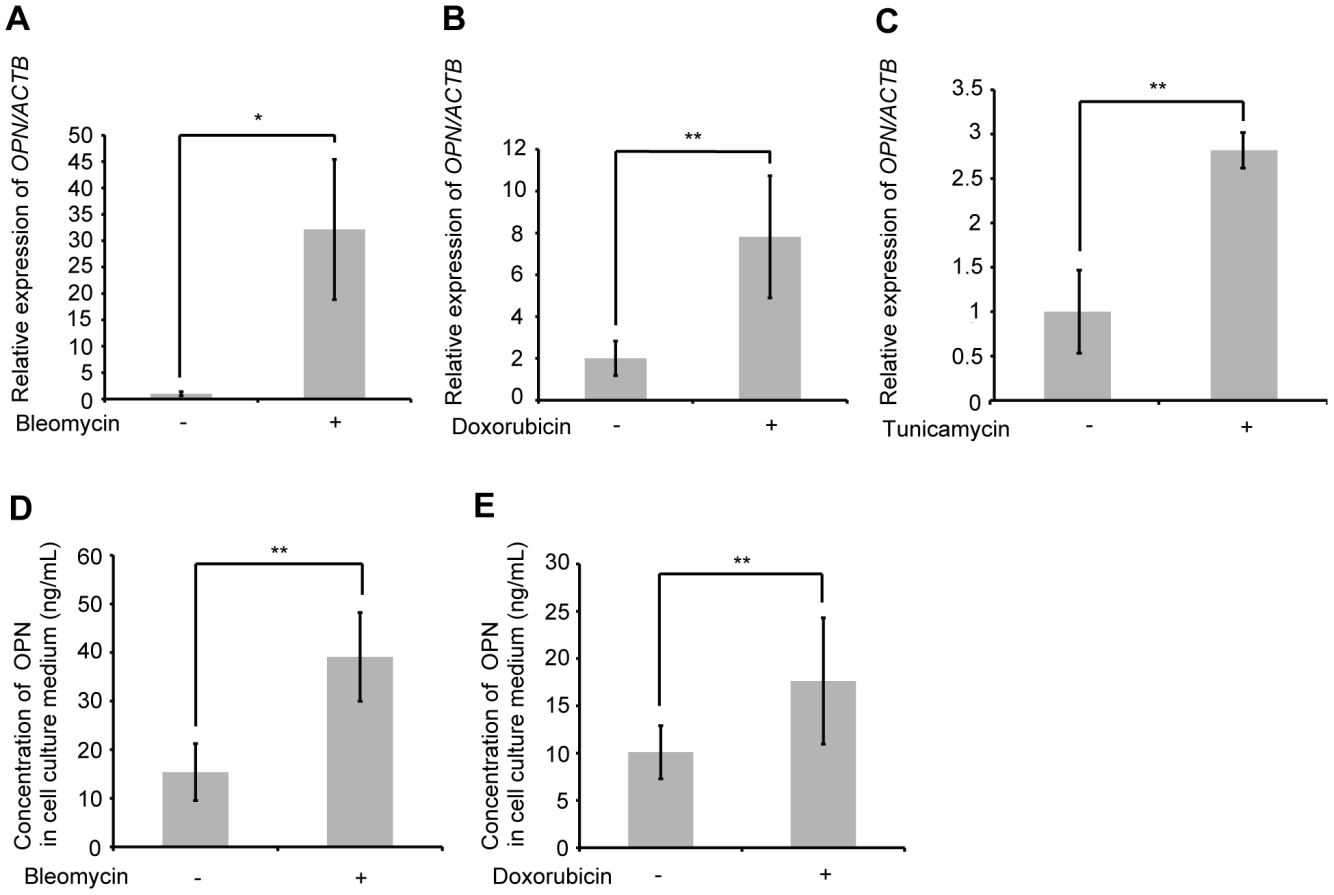
Effect of bleomycin, doxorubicin or tunicamycin on OPN expression in human pulmonary alveolar epithelial cells. (A) HPAEpiC were exposed to 1 µg/mL bleomycin for 48 h. *OPN* mRNA was assessed by qRT-PCR. (B) HPAEpiC were exposed to 50 nM doxorubicin for 48 h. *OPN* mRNA was assessed by qRT-PCR. (C) HPAEpiC were exposed to 0.025 µg/mL tunicamycin for 48 h. *OPN* mRNA was assessed by qRT-PCR. (D) HPAEpiC were exposed to 1 µg/mL bleomycin for 48 h. OPN concentrations in cell culture media were determined by ELISA. (E) HPAEpiC were exposed to 50 nM doxorubicin for 48 h. OPN concentrations in cell culture media were determined by ELISA. Data are presented as mean ± S.E. *, P<0.05; **, P<0.01.

To determine whether bleomycin, doxorubicin and tunicamycin can alter *OPN* mRNA expression in normal human pulmonary alveolar epithelial cells, HPAEpiC were exposed to either 1 µg/mL bleomycin, 50 nM doxorubicin, or 0.025 µg/mL tunicamycin. The expression of *OPN* mRNA was elevated after 48 h exposure to bleomycin, doxorubicin or tunicamycin (Figures 8A, 8B and 8C). Subsequently, we sought to determine whether bleomycin or doxorubicin would induce the production of OPN by HPAEpiC. The accumulation of OPN in HPAEpiC cell culture media was upregulated after 48 h exposure to bleomycin or doxorubicin (Figures 8D and 8E). Our results suggest that these 3 reagents induce OPN production in normal human alveolar epithelial cells as well as in human and murine alveolar epithelial cell lines.

## Discussion

The natural history and pathogenesis of IPF remain poorly understood. Accumulating evidence suggests that IPF may result from the aberrant migration, proliferation and activation of mesenchymal cells provoked when alveolar epithelial cells are subjected to repetitive stress [22]. On the other hand, OPN is a fibrogenic cytokine [6], [7] and its expression is elevated in hyperplastic AEC II of human IPF and murine bleomycin-induced lung fibrosis [7]–[9]. We hypothesized that OPN might play a role in fibrogenesis of the lung and, therefore, studied the mechanism of OPN induction in AEC II and elucidated the functions of OPN in AEC II and lung fibroblasts.

Bleomycin and doxorubicin both cause cellular injury through DNA damage [4], [16]. The present study clearly demonstrates that DNA damage in AEC II caused by bleomycin or doxorubicin induces OPN expression via activation of the ERK-dependent signaling pathway (Figures 1–3 and S1–S7). However, it seems that OPN expression was not directly induced by bleomycin or doxorubicin, because the expression of *OPN* mRNA gradually increased and its induction was observed after 48 h exposure to either bleomycin or doxorubicin. The expression of OPN is induced by various cytokines, such as tumor necrosis factor-α (TNF-α), platelet-derived growth factor, angiotensin II and interleukin-1β [12], [23]-[25]. Thus, it is possible that bleomycin and doxorubicin may have induced cytokines, such as TNF-α, and that those cytokines might, in turn, have induced the expression of OPN.

Patients with familial IPF account for less than 5% of all IPF cases [26]. However, many investigators have studied familial IPF to understand the pathogenesis of IPF, and results suggest that mutations in the *SFTPC* gene (encoding surfactant protein C) and the *SFTPA2* gene (encoding surfactant protein A2) occur in familial IPF. These mutations are likely associated with repetitive injury to alveolar epithelial cells, and cause the unfolding and misfolding of proteins in the ER [17], [18], a potentially major source of stress that could induce pulmonary fibrosis. Tunicamycin inhibits glycoprotein synthesis by inhibition of *N*-linked glycosylation in the ER and thereby induces ER-stress [19], [27]. Therefore, we evaluated tunicamycin, an ER-stress inducer, could induce the expression of OPN in AEC II. The exposure of AEC II to tunicamycin was found to induce the expression of *OPN* mRNA via activation of the ERK-dependent signaling pathway (Figures 4 and S8 - S12). However, the accumulation of OPN in A549 cell culture media was not increased (data not shown). Tunicamycin induces ER stress by inhibiting the first step in the *N*-linked glycosylation of glycoproteins [27]. Moreover, the fact that OPN is an *N*-linked glycoprotein, and that glycosylation is necessary for the secretion of glycoproteins [27], suggests a reason for why the accumulation of OPN in cell culture media was not increased during tunicamycin treatment. To confirm the relationship between ER stress and OPN expression, we investigated the use of another reagent that induces ER stress, namely thapsigargin. Thapsigargin inhibits the Ca^2+^-ATPase in ER and thereby induces ER stress [21]. Thapsigargin induced the expression of *OPN* mRNA in A549 and the accumulation of OPN in A549 cell culture media (Figures 5, S13 and S14). ERK1/2 is strongly activated in the lung tissue of patients during the early stages of IPF [28]. This activation protects AEC II against oxygen-induced DNA damage and apoptosis [29]. According to the results of this study, as well as previous reports, OPN might be expressed as a survival factor for AEC II. We also examined the effect of OPN on AEC II, and have demonstrated here, as well as in a previous study [7], that OPN promotes the proliferation of AEC II (Figure 6). Apoptosis of AEC II is associated with the destruction of alveolar epithelium in IPF [30], [31]. Considering these results, bleomycin-induced OPN may protect AEC II against DNA damage.

Both the migration and accumulation of lung fibroblasts into the alveolar spaces are essential for the development of intra-alveolar fibrosis, a pathologic step in the development of IPF. Therefore, we examined the effect of OPN on lung fibroblasts. The results of this study suggest that OPN promotes the migration of lung fibroblasts (Figure 7), confirming the results of a previous study [6]. Taken together, these results suggest that OPN is a chemoattractant for fibrogenesis of the lung.

In conclusion, we have demonstrated that DNA damage caused by both bleomycin and doxorubicin [4], [16], as well as ER stress caused by tunicamycin [5], induces the expression of OPN in AEC II via activation of the ERK-dependent signaling pathway. The induced OPN promotes the proliferation of AEC II and migration of lung fibroblasts. Moreover, proliferation of AEC II by OPN induces further OPN production. Based on these results, OPN might function as a survival factor for AEC II. However, excessive OPN might be associated with the abnormal repair that causes fibrogenesis. To better understand the relationship between OPN and fibrogenesis of the lung, further studies using in vivo murine models of lung fibrosis are needed.

## Supporting Information

Figure S1 Bleomycin induced Erk1/2 phospholylation in MLE12. MLE12 were exposed to 10 µg/mL bleomycin or PBS for 36 h. Western blotting was used to detect phospho-specific Erk1/2 and Erk1/2.
(TIF)

Figure S2 ERK1/2 inhibitors inhibited Erk1/2 phospholylation after beomycin exposure in MLE12. (A) MLE12 were pretreated with PD98059 (50 µM) or DMSO for 1 h, and subsequently exposed to 10 µg/mL bleomycin for 48 h. Western blotting was used to detect phospho-specific Erk1/2 and Erk1/2. (B) MLE12 were pretreated with U0126 (50 µM) or DMSO for 1 h, and subsequently exposed to 10 µg/mL bleomycin for 48 h. Western blotting was used to detect phospho-specific Erk1/2 and Erk1/2.
(TIF)

Figure S3 ERK1/2 inhibitors reduced the expression of *Opn* mRNA after bleomycin exposure in MLE12. (A) MLE12 were pretreated with PD98059 (50 µM) or DMSO for 1 h, and subsequently exposed to 10 µg/mL bleomycin or PBS for 48 h. *Opn* mRNA was assessed by qRT-PCR. (B) MLE12 were pretreated with U0126 (50 µM) or DMSO for 1 h, and subsequently exposed to 10 µg/mL bleomycin or PBS for 48 h. *Opn* mRNA was assessed by qRT-PCR. Data are presented as mean ± S.E. *, P<0.05; **, P<0.01.
(TIF)

Figure S4 Doxorubicin induced the expression of *Opn* mRNA in MLE12. MLE12 were exposed to 50 nM doxorubicin or PBS for 48 h. *Opn* mRNA was assessed by qRT-PCR. Data are presented as mean ± S.E. *, P<0.05; **, P<0.01.
(TIF)

Figure S5 Doxorubicin induced Erk1/2 phospholylation in MLE12. MLE12 were exposed to 50 nM doxorubicin or PBS for 36 h. Western blotting was used to detect phospho-specific Erk1/2 and Erk1/2.
(TIF)

Figure S6 ERK1/2 inhibitors inhibited Erk1/2 phospholylation after doxorubicin exposure in MLE12. (A) MLE12 were pretreated with PD98059 (50 µM) or DMSO for 1 h, and subsequently exposed to 50 nM doxorubicin for 48 h. Western blotting was used to detect phospho-specific Erk1/2 and Erk1/2. (B) MLE12 were pretreated with U0126 (50 µM) or DMSO for 1 h, and subsequently exposed to 50 nM doxorubicin for 48 h. Western blotting was used to detect phospho-specific Erk1/2 and Erk1/2.
(TIF)

Figure S7 ERK1/2 inhibitors reduced the expression of *Opn* mRNA after doxorubicin exposure in MLE12. (A) MLE12 were pretreated with PD98059 (50 µM) or DMSO for 1 h, and subsequently exposed to 50 nM doxorubicin or PBS for 48 h. *Opn* mRNA was assessed by qRT-PCR. (B) MLE12 were pretreated with U0126 (50 µM) or DMSO for 1 h, and subsequently exposed to 50 nM doxorubicin or PBS for 48 h. *Opn* mRNA was assessed by qRT-PCR. Data are presented as mean ± S.E. *, P<0.05; **, P<0.01.
(TIF)

Figure S8 Tunicamycin induced the expression of BiP protein in MLE12. MLE12 were exposed to 0.025 µg/mL tunicamycin or DMSO for 48 h. Western blotting was used to detect BiP and β-actin.
(TIF)

Figure S9 Tunicamycin induced the expression of *Opn* mRNA in MLE12. MLE12 were exposed to 0.025 µg/mL tunicamycin or DMSO for 48 h. *Opn* mRNA was assessed by qRT-PCR. Data are presented as mean ± S.E. *, P<0.05; **, P<0.01.
(TIF)

Figure S10 Tunicamycin induced Erk1/2 phospholylation in MLE12. MLE12 were exposed to 0.025 µg/mL tunicamycin or DMSO for 36 h. Western blotting was used to detect phospho-specific Erk1/2 and Erk1/2.
(TIF)

Figure S11 ERK1/2 inhibitors inhibited Erk1/2 phospholylation after tunicamycin exposure in MLE12. (A) MLE12 were pretreated with PD98059 (50 µM) or DMSO for 1 h, and subsequently exposed to 0.025 µg/mL tunicamycin for 48 h. Western blotting was used to detect phospho-specific Erk1/2 and Erk1/2. (B) MLE12 were pretreated with U0126 (50 µM) or DMSO for 1 h, and subsequently exposed to 0.025 µg/mL tunicamycin for 48 h. Western blotting was used to detect phospho-specific Erk1/2 and Erk1/2.
(TIF)

Figure S12 ERK1/2 inhibitors reduced the expression of *Opn* mRNA after tunicamycin exposure in MLE12. (A) MLE12 were pretreated with PD98059 (50 µM) or DMSO for 1 h, and subsequently exposed to 0.025 µg/mL tunicamycin or DMSO for 48 h. *Opn* mRNA was assessed by qRT-PCR. (B) MLE12 were pretreated with U0126 (50 µM) or DMSO for 1 h, and subsequently exposed to 0.025 µg/mL tunicamycin or DMSO for 48 h. *Opn* mRNA was assessed by qRT-PCR. Data are presented as mean ± S.E. *, P<0.05; **, P<0.01.
(TIF)

Figure S13 Thapsigargin induced the expression of BiP protein in MLE12. MLE12 were exposed to 5 nM thapsigargin or DMSO for 48 h. Western blotting was used to detect BiP and β-actin.
(TIF)

Figure S14 Thapsigargin induced the expression of *Opn* mRNA in MLE12. MLE12 were exposed to 5 nM thapsigargin or DMSO for 48 h. *Opn* mRNA was assessed by qRT-PCR. Data are presented as mean ± S.E. *, P<0.05; **, P<0.01.
(TIF)

## References

[1] Raghu G, Collard HR, Egan JJ, Martinez FJ, Behr J, et al (2011) An official ATS/ERS/JRS/ALAT statement: idiopathic pulmonary fibrosis: evidence-based guidelines for diagnosis and management. Am J Respir Crit Care Med 183: 788–824. 21471066 10.1164/rccm.2009-040GL PMC5450933

[2] Kuhn C, McDonald JA (1991) The roles of the myofibroblast in idiopathic pulmonary fibrosis: ultrastructural and immunohistochemical features of sites of active extracellular matrix synthesis. Am J Pathol 138: 1257–1265. 2024710 PMC1886011

[3] Seiman M, Pardo A (2006) Role of epithelial cells in idiopathic pulmonary fibrosis: from innocent targets to serial killers. Proc Am Thorac Soc 3: 364–372. 16738202 10.1513/pats.200601-003TK

[4] Mishra A, Doyle NA, Martin WJ 2cnd (2000) Bleomycin-mediated pulmonary toxicity: evidence for a p53-mediated response. Am J Respir Cell Mol Biol 22: 543–549. 10783125 10.1165/ajrcmb.22.5.3851

[5] Lawson WE, Cheng DS, Degryse AL, Tanjore H, Polosukhin VV, et al (2011) Endoplasmic reticulum stress enhances fibrotic remodeling in the lungs. Proc Natl Acad Sci U S A 108: 10562–10567. 21670280 10.1073/pnas.1107559108 PMC3127925

[6] Takahashi F, Takahashi K, Okazaki T, Maeda K, Ienaga H, et al (2001) Role of osteopontin in the pathogenesis of bleomycin-induced pulmonary fibrosis. Am J Respir Cell Mol Biol 24: 264–271. 11245625 10.1165/ajrcmb.24.3.4293

[7] Pardo A, Gibson K, Cisneros J, Richards TJ, Yang Y, et al (2005) Up-regulation and profibrotic role of osteopontin in human idiopathic pulmonary fibrosis. PLoS Med 2: e251. 16128620 10.1371/journal.pmed.0020251 PMC1198037

[8] Kelly MM, Leigh R, Gilpin SE, Cheng E, Martin GE, et al (2006) Cell-specific gene expression in patients with usual interstitial pneumonia. Am J Respir Crit Care Med 174: 557–565. 16728711 10.1164/rccm.200510-1648OC

[9] Berman JS, Serlin D, Li X, Whitley G, Hayes J, et al (2004) Altered bleomycin-induced lung fibrosis in osteopontin-deficient mice. Am J Physiol Lung Cell Mol Physiol 286: L1311–L1318. 14977630 10.1152/ajplung.00394.2003

[10] Day RM, Yang Y, Suzuki YJ, Stevens J, Pathi R, et al (2001) Bleomycin upregulates gene expression of angiotensin-converting enzyme via mitogen-activated protein kinase and early growth response 1 transcription factor. Am J Respir Cell Mol Biol 25: 613–619. 11713104 10.1165/ajrcmb.25.5.4521

[11] Matsuoka H, Arai T, Mori M, Goya S, Kida H, et al (2002) A p38 MAPK inhibitor, FR-167653, ameliorates murine bleomycin-induced pulmonary fibrosis. Am J Physiol Lung Cell Mol Physiol 283: L103–L112. 12060566 10.1152/ajplung.00187.2001

[12] Xie Z, Singh M, Singh K (2004) ERK1/2 and JNKs, but not p38 kinase, are involved in reactive oxygen species-mediated induction of osteopontin gene expression by angiotensin II and interleukin-1β in adult rat cardiac fibroblasts. J Cell Physiol 198: 399–407. 14755545 10.1002/jcp.10419

[13] Hong SY, Jeon YM, Lee HJ, Kim JG, Baek JA, et al (2010) Activation of RhoA and FAK induces ERK-mediated osteopontin expression in mechanical force-subjected periodontal ligament fibroblasts. Mol Cell Biochem 335: 263–272. 19798549 10.1007/s11010-009-0276-1

[14] Alessi DR, Cuenda A, Cohen P, Dudley DT, Saltiel AR (1995) PD98059 is a specific inhibitor of the activation of mitogen-activated protein kinase kinase in vitro and in vivo. J Biol Chem 270: 27489–27494. 7499206 10.1074/jbc.270.46.27489

[15] Favata MF, Horiuchi KY, Manos EJ, Daulerio AJ, Stradley DA, et al (1998) Identification of a novel inhibitor of mitogen-activated protein kinase kinase. J Biol Chem 273: 18623–18632. 9660836 10.1074/jbc.273.29.18623

[16] Tewey KM, Rowe TC, Yang L, Halligan BD, Liu LF (1984) Adriamycin-induced DNA damage mediated by mammalian DNA topoisomerase II. Science 226: 466–468. 6093249 10.1126/science.6093249

[17] Nogee LM, Dunbar AE 3rd, Wert SE, Askin F, Hamvas A, et al (2001) A mutation in the surfactant protein C gene associated with familial interstitial lung disease. N Engl J Med 344: 573–579. 11207353 10.1056/NEJM200102223440805

[18] Maitra M, Cano CA, Garcia CK (2012) Mutant surfactant A2 proteins associated with familial pulmonary fibrosis and lung cancer induce TGF-β1 secretion. Proc Natl Acad Sci U S A 109: 21064–21069. 23223528 10.1073/pnas.1217069110 PMC3529022

[19] Leleu X, Xu L, Jia X, Sacco A, Farag M, et al (2009) Endoplasmic reticulum stress is a target for therapy in Waldenstrom macroglobulinemia. Blood 113: 626–634. 18981296 10.1182/blood-2007-10-116848

[20] Kaufman RJ (1999) Stress signaling from the lumen of the endoplasmic reticulum: coordination of gene transcriptional and translational controls. Genes Dev 13: 1211–1233. 10346810 10.1101/gad.13.10.1211

[21] Wictome M, Henderson I, Lee AG, East JM (1992) Mechanism of inhibition of the calcium pump of sarcoplasmic reticulum by thapsigargin. Biochem J 283: 525–529. 1533513 10.1042/bj2830525 PMC1131067

[22] Adamson IY, Young L, Bowden DH (1988) Relationship of alveolar epithelial injury and repair to the induction of pulmonary fibrosis. Am J Pathol 130: 377–383. 3341452 PMC1880524

[23] Miyazaki Y, Tashiro T, Higuchi Y, Setoguchi M, Yamamoto S, et al (1995) Expression of osteopontin in a macrophage cell line and in transgenic mice with pulmonary fibrosis resulting from the lung expression of a tumor necrosis factor-α transgene. Ann N Y Acad Sci 760: 334–341. 7785911 10.1111/j.1749-6632.1995.tb44651.x

[24] Wang X, Louden C, Ohlstein EH, Stadel JM, Gu JL, et al (1996) Osteopontin expression in platelet-derived growth factor-stimulated vascular smooth muscle cells and carotid artery after balloon angioplasty. Arterioscler Thromb Vasc Biol 16: 1365–1372. 8911275 10.1161/01.atv.16.11.1365

[25] Serlin DM, Kuang PP, Subramanian M, O'Regan A, Li X, et al (2006) Interleukin-1β induces osteopontin expression in pulmonary fibroblasts. J Cell Biochem 97: 519–529. 16211580 10.1002/jcb.20661

[26] Hodgson U, Laitinen T, Tukiainen P (2002) Nationwide prevalence of sporadic and familial idiopathic pulmonary fibrosis: evidence of founder effect among multiplex families in Finland. Thorax 57: 338–342. 11923553 10.1136/thorax.57.4.338 PMC1746288

[27] Björkman U, Ekholm R (1982) Effect of tunicamycin on thyroglobulin secretion. Eur J Biochem 125: 585–591. 7117256 10.1111/j.1432-1033.1982.tb06723.x

[28] Yoshida K, Kuwano K, Hagimoto N, Watanabe K, Matsuba T, et al (2002) MAP kinase activation and apoptosis in lung tissues from patients with idiopathic pulmonary fibrosis. J Pathol 198: 388–396. 12375272 10.1002/path.1208

[29] Buckley S, Driscoll B, Barsky L, Weinberg K, Anderson K, et al (1999) ERK activation protects against DNA damage and apoptosis in hyperoxic rat AEC2. Am J Physiol Lung Cell Mol Physiol 277: L159–L166. 10.1152/ajplung.1999.277.1.L159 10409243

[30] Barbas-Filho JV, Ferreira MA, Sesso A, Kairalla RA, Carvalho CR, et al (2001) Evidence of type II pneumocyte apoptosis in the pathogenesis of idiopathic pulmonary fibrosis (IFP)/usual interstitial pneumonia (UIP). J Clin Pathol 54: 132–138. 11215282 10.1136/jcp.54.2.132 PMC1731356

[31] Lee VY, Schroedl C, Brunelle JK, Buccellato LJ, Akinci OI, et al (2005) Bleomycin induces alveolar epithelial cell death through JNK-dependent activation of the mitochondrial death pathway. Am J Physiol Lung Cell Mol Physiol 289: L524–L528. 10.1152/ajplung.00340.2004 16148050

