# Supplementary figures and images for: Cell Stress Induces Upregulation of Osteopontin via the ERK Pathway in Type II Alveolar Epithelial Cells
Source: PLoS One. 2014 Jun 25;9(6):e100106. doi: 10.1371/journal.pone.0100106 (PMC4070890; doi:10.1371/journal.pone.0100106)

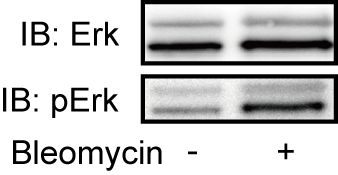

Supplement: Figure S1 — Bleomycin induced Erk1/2 phospholylation in MLE12. MLE12 were exposed to 10 µg/mL bleomycin or PBS for 36 h. Western blotting was used to detect phospho-specific Erk1/2 and Erk1/2. (TIF) [file pone.0100106.s001.tif]

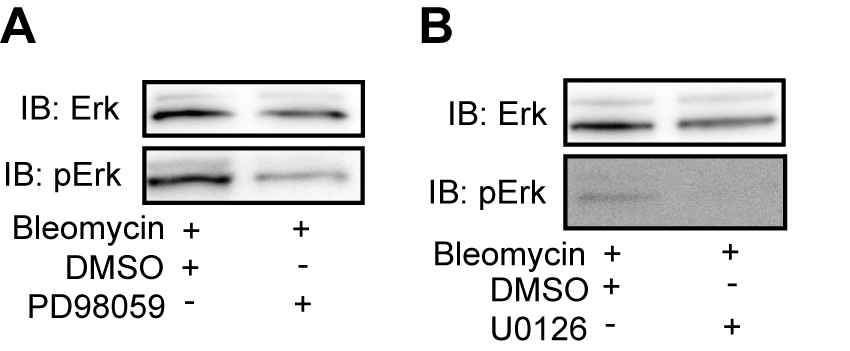

Supplement: Figure S2 — ERK1/2 inhibitors inhibited Erk1/2 phospholylation after beomycin exposure in MLE12. (A) MLE12 were pretreated with PD98059 (50 µM) or DMSO for 1 h, and subsequently exposed to 10 µg/mL bleomycin for 48 h. Western blotting was used to detect phospho-specific Erk1/2 and Erk1/2. (B) MLE12 were pretreated with U0126 (50 µM) or DMSO for 1 h, and subsequently exposed to 10 µg/mL bleomycin for 48 h. Western blotting was used to detect phospho-specific Erk1/2 and Erk1/2. (TIF) [file pone.0100106.s002.tif]

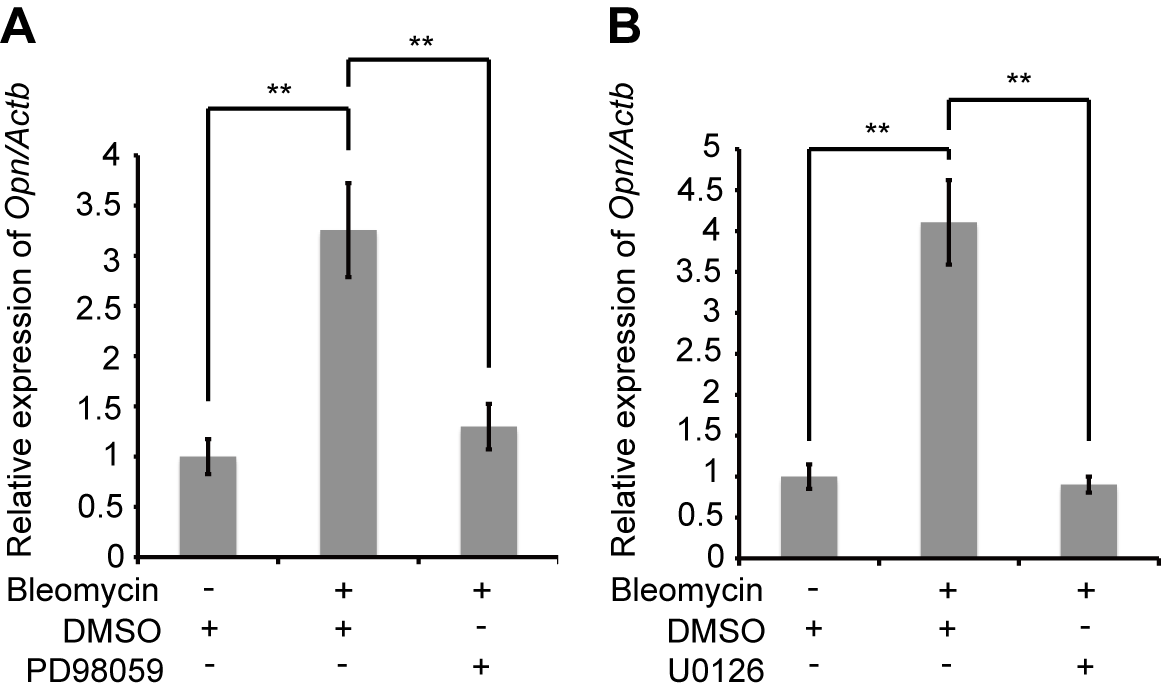

Supplement: Figure S3 — ERK1/2 inhibitors reduced the expression of Opn mRNA after bleomycin exposure in MLE12. (A) MLE12 were pretreated with PD98059 (50 µM) or DMSO for 1 h, and subsequently exposed to 10 µg/mL bleomycin or PBS for 48 h. Opn mRNA was assessed by qRT-PCR. (B) MLE12 were pretreated with U0126 (50 µM) or DMSO for 1 h, and subsequently exposed to 10 µg/mL bleomycin or PBS for 48 h. Opn mRNA was assessed by qRT-PCR. Data are presented as mean ± S.E. *, P<0.05; **, P<0.01. (TIF) [file pone.0100106.s003.tif]

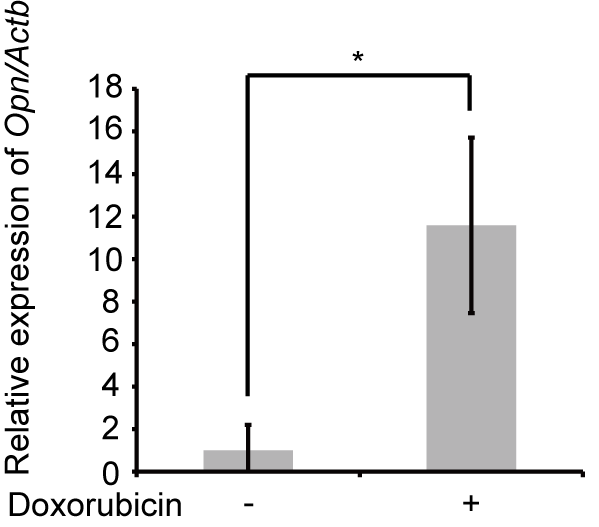

Supplement: Figure S4 — Doxorubicin induced the expression of Opn mRNA in MLE12. MLE12 were exposed to 50 nM doxorubicin or PBS for 48 h. Opn mRNA was assessed by qRT-PCR. Data are presented as mean ± S.E. *, P<0.05; **, P<0.01. (TIF) [file pone.0100106.s004.tif]

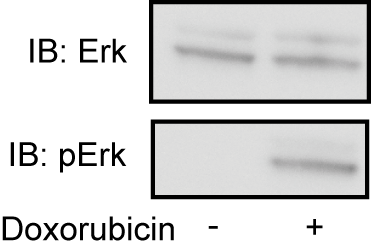

Supplement: Figure S5 — Doxorubicin induced Erk1/2 phospholylation in MLE12. MLE12 were exposed to 50 nM doxorubicin or PBS for 36 h. Western blotting was used to detect phospho-specific Erk1/2 and Erk1/2. (TIF) [file pone.0100106.s005.tif]

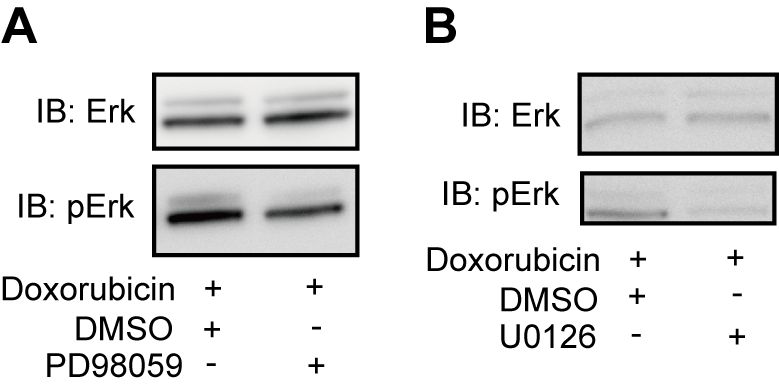

Supplement: Figure S6 — ERK1/2 inhibitors inhibited Erk1/2 phospholylation after doxorubicin exposure in MLE12. (A) MLE12 were pretreated with PD98059 (50 µM) or DMSO for 1 h, and subsequently exposed to 50 nM doxorubicin for 48 h. Western blotting was used to detect phospho-specific Erk1/2 and Erk1/2. (B) MLE12 were pretreated with U0126 (50 µM) or DMSO for 1 h, and subsequently exposed to 50 nM doxorubicin for 48 h. Western blotting was used to detect phospho-specific Erk1/2 and Erk1/2. (TIF) [file pone.0100106.s006.tif]

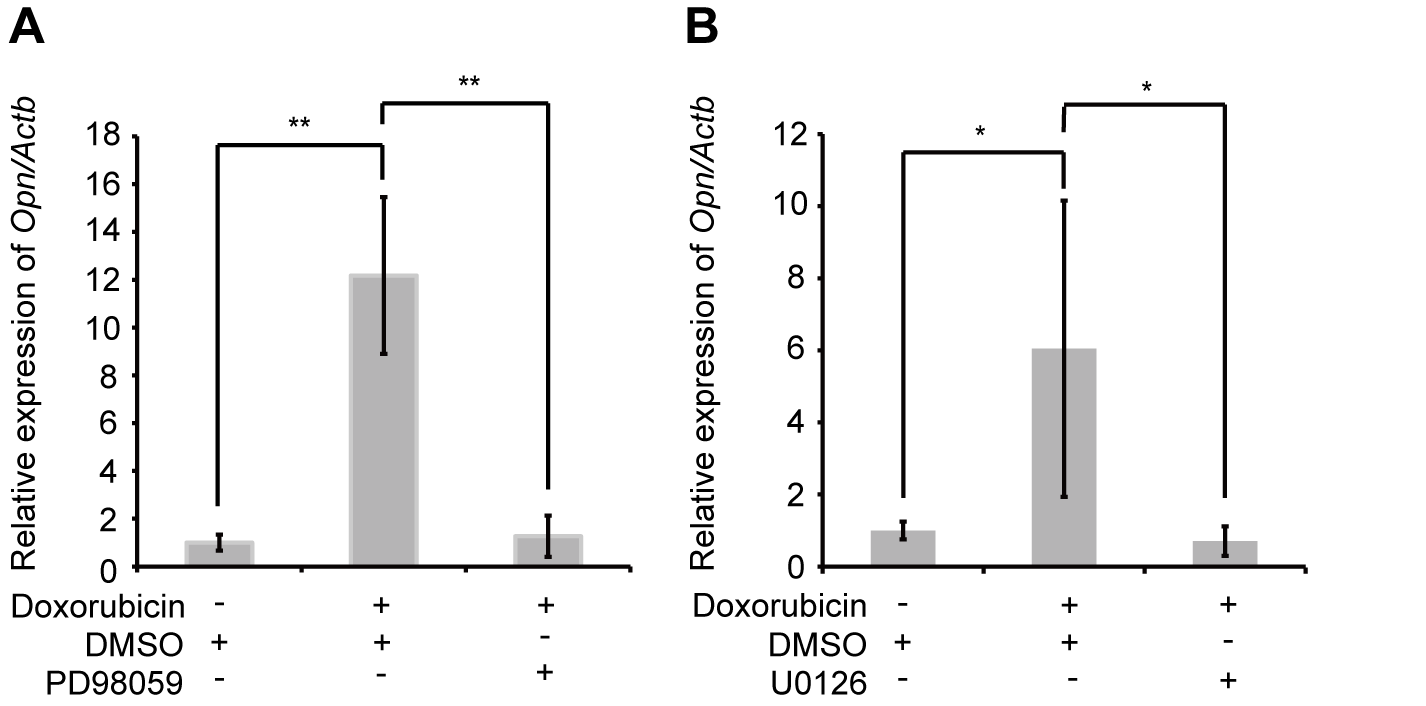

Supplement: Figure S7 — ERK1/2 inhibitors reduced the expression of Opn mRNA after doxorubicin exposure in MLE12. (A) MLE12 were pretreated with PD98059 (50 µM) or DMSO for 1 h, and subsequently exposed to 50 nM doxorubicin or PBS for 48 h. Opn mRNA was assessed by qRT-PCR. (B) MLE12 were pretreated with U0126 (50 µM) or DMSO for 1 h, and subsequently exposed to 50 nM doxorubicin or PBS for 48 h. Opn mRNA was assessed by qRT-PCR. Data are presented as mean ± S.E. *, P<0.05; **, P<0.01. (TIF) [file pone.0100106.s007.tif]

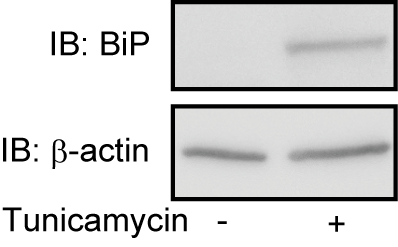

Supplement: Figure S8 — Tunicamycin induced the expression of BiP protein in MLE12. MLE12 were exposed to 0.025 µg/mL tunicamycin or DMSO for 48 h. Western blotting was used to detect BiP and β-actin. (TIF) [file pone.0100106.s008.tif]

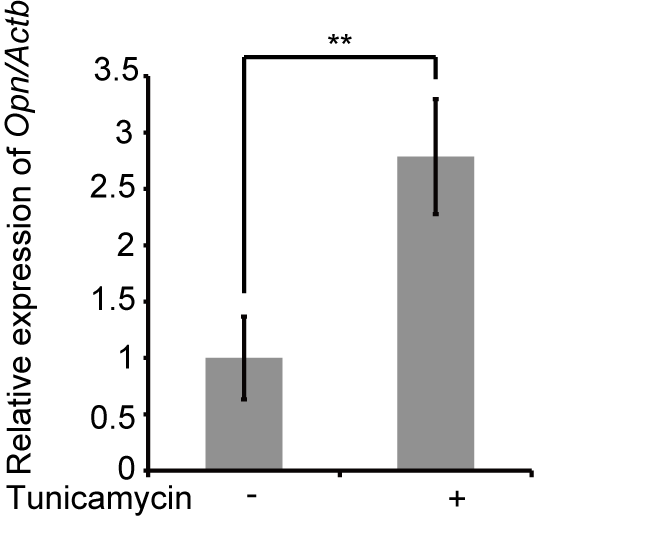

Supplement: Figure S9 — Tunicamycin induced the expression of Opn mRNA in MLE12. MLE12 were exposed to 0.025 µg/mL tunicamycin or DMSO for 48 h. Opn mRNA was assessed by qRT-PCR. Data are presented as mean ± S.E. *, P<0.05; **, P<0.01. (TIF) [file pone.0100106.s009.tif]

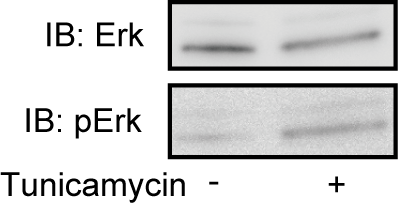

Supplement: Figure S10 — Tunicamycin induced Erk1/2 phospholylation in MLE12. MLE12 were exposed to 0.025 µg/mL tunicamycin or DMSO for 36 h. Western blotting was used to detect phospho-specific Erk1/2 and Erk1/2. (TIF) [file pone.0100106.s010.tif]

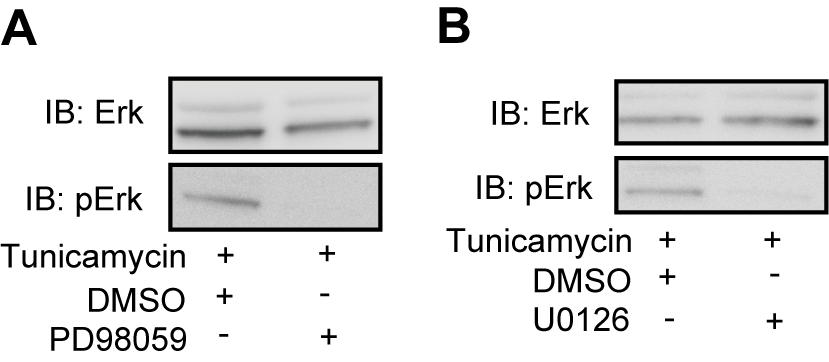

Supplement: Figure S11 — ERK1/2 inhibitors inhibited Erk1/2 phospholylation after tunicamycin exposure in MLE12. (A) MLE12 were pretreated with PD98059 (50 µM) or DMSO for 1 h, and subsequently exposed to 0.025 µg/mL tunicamycin for 48 h. Western blotting was used to detect phospho-specific Erk1/2 and Erk1/2. (B) MLE12 were pretreated with U0126 (50 µM) or DMSO for 1 h, and subsequently exposed to 0.025 µg/mL tunicamycin for 48 h. Western blotting was used to detect phospho-specific Erk1/2 and Erk1/2. (TIF) [file pone.0100106.s011.tif]

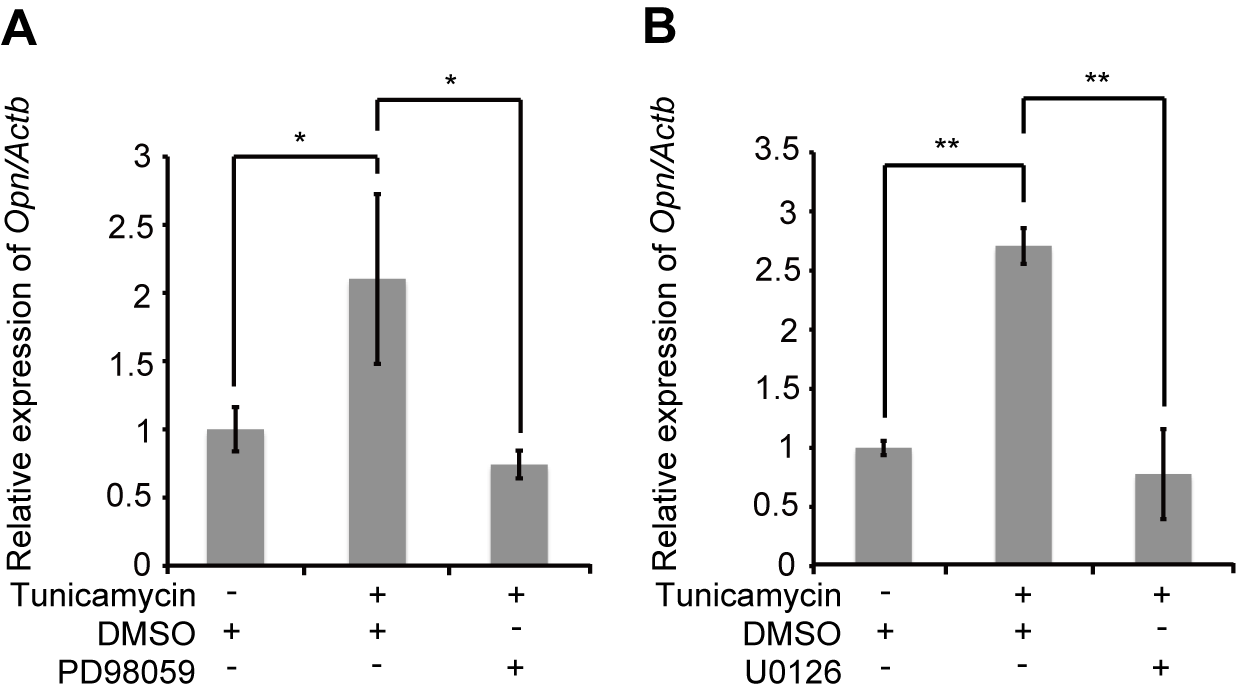

Supplement: Figure S12 — ERK1/2 inhibitors reduced the expression of Opn mRNA after tunicamycin exposure in MLE12. (A) MLE12 were pretreated with PD98059 (50 µM) or DMSO for 1 h, and subsequently exposed to 0.025 µg/mL tunicamycin or DMSO for 48 h. Opn mRNA was assessed by qRT-PCR. (B) MLE12 were pretreated with U0126 (50 µM) or DMSO for 1 h, and subsequently exposed to 0.025 µg/mL tunicamycin or DMSO for 48 h. Opn mRNA was assessed by qRT-PCR. Data are presented as mean ± S.E. *, P<0.05; **, P<0.01. (TIF) [file pone.0100106.s012.tif]

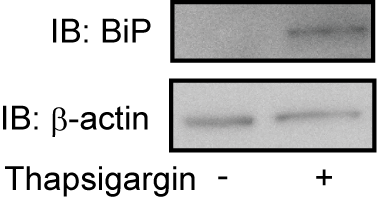

Supplement: Figure S13 — Thapsigargin induced the expression of BiP protein in MLE12. MLE12 were exposed to 5 nM thapsigargin or DMSO for 48 h. Western blotting was used to detect BiP and β-actin. (TIF) [file pone.0100106.s013.tif]

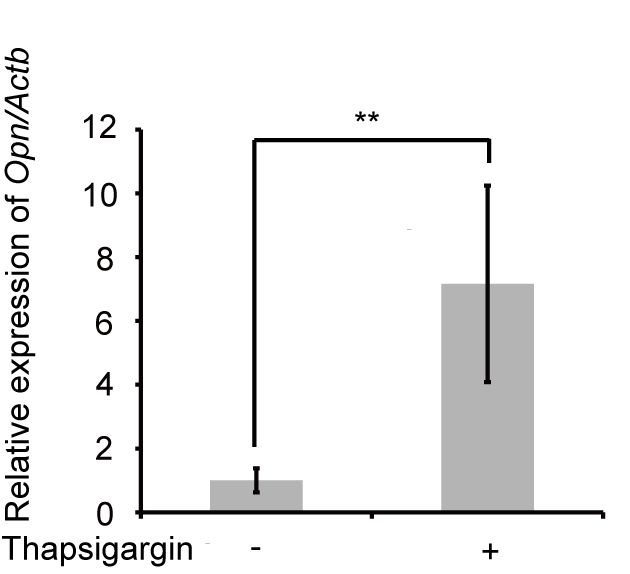

Supplement: Figure S14 — Thapsigargin induced the expression of Opn mRNA in MLE12. MLE12 were exposed to 5 nM thapsigargin or DMSO for 48 h. Opn mRNA was assessed by qRT-PCR. Data are presented as mean ± S.E. *, P<0.05; **, P<0.01. (TIF) [file pone.0100106.s014.tif]
